# A Synergic Potential of Antimicrobial Peptides against *Pseudomonas syringae* pv. *actinidiae*

**DOI:** 10.3390/molecules26051461

**Published:** 2021-03-08

**Authors:** Nuno Mariz-Ponte, Laura Regalado, Emil Gimranov, Natália Tassi, Luísa Moura, Paula Gomes, Fernando Tavares, Conceição Santos, Cátia Teixeira

**Affiliations:** 1 Biology Department, Faculty of Science, University of Porto (FCUP), 4169-007 Porto, Portugal; up201604469@fc.up.pt (L.R.); up201804355@fc.up.pt (E.G.); ftavares@fc.up.pt (F.T.); csantos@fc.up.pt (C.S.); 2 LAQV-REQUIMTE, Biology Department, Faculty of Science (FCUP), University of Porto, 4169-007 Porto, Portugal; 3 CIBIO—Research Centre in Biodiversity and Genetic Resources, In-BIO-Associate Laboratory, Microbial Diversity and Evolution Group, University of Porto (UP), 4485-661 Vairão, Portugal; 4 LAQV-REQUIMTE, Department of Chemistry and Biochemistry, Faculty of Sciences (FCUP), University of Porto, 4169-007 Porto, Portugal; natalia.tcpf@fc.up.pt (N.T.); pgomes@fc.up.pt (P.G.); catia.teixeira@fc.up.pt (C.T.); 5 CISAS—Centre for Research and Development in Agrifood Systems and Sustainability, Instituto Politécnico de Viana do Castelo, 4900-347 Viana do Castelo, Portugal; luisamoura@esa.ipvc.pt

**Keywords:** 3.1, *Actinidia* sp., antimicrobial peptides, bacterial canker of kiwifruit, BP100, CA-M, D4E1, Dhvar-5, *Pseudomonas syringae* pv. *actinidiae*, RW-BP100

## Abstract

*Pseudomonas syringae* pv. *actinidiae* (Psa) is the pathogenic agent responsible for the bacterial canker of kiwifruit (BCK) leading to major losses in kiwifruit productions. No effective treatments and measures have yet been found to control this disease. Despite antimicrobial peptides (AMPs) having been successfully used for the control of several pathogenic bacteria, few studies have focused on the use of AMPs against Psa. In this study, the potential of six AMPs (BP100, RW-BP100, CA-M, 3.1, D4E1, and Dhvar-5) to control Psa was investigated. The minimal inhibitory and bactericidal concentrations (MIC and MBC) were determined and membrane damaging capacity was evaluated by flow cytometry analysis. Among the tested AMPs, the higher inhibitory and bactericidal capacity was observed for BP100 and CA-M with MIC of 3.4 and 3.4–6.2 µM, respectively and MBC 3.4–10 µM for both. Flow cytometry assays suggested a faster membrane permeation for peptide 3.1, in comparison with the other AMPs studied. Peptide mixtures were also tested, disclosing the high efficiency of BP100:3.1 at low concentration to reduce Psa viability. These results highlight the potential interest of AMP mixtures against Psa, and 3.1 as an antimicrobial molecule that can improve other treatments in synergic action.

## 1. Introduction

Bacterial canker of kiwifruit (BCK) is globally destroying many orchards of *Actinidia deliciosa* and *A. chinensis*, generating dramatic economic losses to producers [1,2]. For that reason, BCK is considered the most important disease of kiwifruit orchards, *Pseudomonas syringae* pv. *actinidiae* (Psa) being the etiologic agent of this disease [1,3,4]. The taxonomy of this gram-negative bacteria is complex due to its physiological and genetic diversity, and because of successive reclassifications, it currently aggregates the biovars 1, 2, 3, 5, and 6 based on molecular and biochemical differences [5,6,7,8]. The biovar 3 (Psa3) is the most virulent and the most disseminated [9,10,11]. Symptoms in the hosts are visible from late winter to late autumn and appear in different plant organs, such as leaves with necrotic points rounded by a chlorotic yellowish halo, bacterial ooze production, dried branches, and necrosis in buds, twigs, and flowers [1,12]. Due to its severity and the absence of efficient control measures, Psa, considered a quarantine pathogen, was in 2020 added to the List of Emergency Measures by the European Plant Protection Organization (EPPO [13]).

The currently available preventive, chemical, and biological control measures for BCK [14] remain inefficient to stop Psa pandemic spread and maintain the required high productivity of kiwifruit orchards [15,16]. The use of antibiotics in orchards (e.g., streptomycin or kasugamycin), while still adopted under specific circumstances in some kiwi-producing countries such as the USA, New Zealand, and China [17,18], is increasingly being restricted in other countries including in the European Union [19]. Furthermore, several studies support that the use of antibiotics to control phytopathogenic bacteria represents an inefficient long-term strategy, considering consumers’ and environmental health. Particularly, the wide use of antibiotics in agriculture may potentiate acquired multi-resistance to conventional antibiotics in targeted and untargeted bacteria with associated risks for animals and human health [19]. 

Psa antibiotic-resistant strains were first found in the late 1980s in Japanese bacterial isolates from kiwifruit plants routinely exposed to streptomycin (aminoglycoside antibiotic), and later identified in other countries, such as Korea and New Zealand [14,15,20]. In New Zealand, streptomycin-resistant strains were identified in 2015 (a few years after the commercial antibiotic usage), supporting rapid resistance acquisition, which compromises antibiotic efficiency, besides the associated risks to human and environmental health. Kasugamicynin is authorized in New Zealand, and despite that no resistant strains as of yet have been identified, it must be stressed that this is a more recent antibiotic from the aminoglycoside family like streptomycin [14].

The main routine disease treatments to control BCK are based and copper (Cu) formulations [21,22]. Although some positive effects on controlling BCK progression have been reported, these are not systemic treatments, but rather mitigation measures [16,23]. Moreover, in soils contaminated with Cu, a decrease in the soil’s community diversity was observed, including beneficial microorganisms [24]. Additionally, Psa strains with acquired resistance to Cu were already found in New Zealand [14,25], and Hu et al. [26] also suggested that excess of copper in agricultural soils promotes the cross acquisition of antibiotic resistance. This confluence of factors makes research into novel efficient and sustainable strategies a priority to control Psa. Among the several innovative strategies that have been proposed as short-term alternatives to control Psa [27,28,29,30], namely the use of bacteriophages [31], essential oils, and plants’ metabolites [32,33,34], the antimicrobial peptides (AMPs) are probably the most consensual solution towards a more sustainable and safer agriculture [32,33,34].

AMPs are constituted by short amino acids (aa) chains, normally between 10 to 100 aa arranged in β-sheet, α-helix, or loop, and with broad range of antimicrobial activity [35]. Over the last 30 years, the pressure to replace the conventional antibiotics in the front line of treatments enhanced the knowledge on AMPs [36]. AMPs are naturally produced by a large diversity of organisms as protection against pathogen attacks and competition [37]. More than 2000 AMP molecules from biological reservoirs were already identified [38] and synthetic AMPs based on natural AMPs have been designed to improve its microbial activity and refine target specificity [39]. Different mechanisms of antimicrobial action have been hypothesized for AMPs, with membrane destabilization being the main function [38]. Regarding gram-negative bacteria, cationic AMPs are attracted to the negatively charged lipopolysaccharides (LPS) of the outer membrane, and hydrogen bonds are established between arginine and lysine and the phosphate groups from lipids, leading to a critical accumulation of AMPs [40], which then move from the outer membrane surface and exert a high tension that culminates in membrane rupture and consequent cell death [41]. Other AMPs exert their action through membrane disorder mechanisms promoting the flipflop of phospholipids, with consequent membrane depolarization leading to bactericidal effects [40,42]. Alternative mechanisms of AMPs action are characterized by membrane permeabilization and cell function regulation, such as inhibition of DNA transcription, mRNA synthesis, peptidoglycan synthesis, protein synthesis, and activity [43]. Furthermore, the low toxicity for host species [44,45] and the limited bacterial resistance acquired [40,46] makes the use of AMPs for plant protection as one of the most promising practices towards a sustainable agriculture [15,47]. 

In the last few years, diverse AMPs have been reported to possess a high bactericidal activity against several phytopathogenic bacteria [48,49,50,51,52]. The plant pathogenic *Pseudomonas* genus has obtained some attention as a target for the development of new treatments based on AMPs [53,54,55,56,57]. Particularly, different AMPs were found to have a bactericidal effect on Psa by disrupting its membrane, at different ranges of concentration [30,31,32]. BP100 is one of the most tested AMPs, since Badosa et al. [58] designed and demonstrated its high potential to control phytopathogenic bacteria. The bioactivity of this AMP has been demonstrated against *Erwinia amylovora* [48], *Xyllela fastidiosa* [51], *Dickeya chrysanthemi* [59] and also tested against Psa [29]. Due to the potential of this peptide, several analogs were designed and tested on pathogenic bacteria, such as RW-BP100 [60]. This peptide was reported only for clinical pathogenic bacteria, demonstrating high efficiency on gram-negative bacteria, namely *Acinetobacter baumannii* [61] and *Escherichia coli* [50]. D4E1 peptide emerged as a strong antifungal AMP [62], although its efficiency was also proved in phytopathogenic bacteria such as *Xanthomonas campestris* pv. *malvacearum* and *P. syringae* pv. *tabaci* [53]. It was also expressed in transgenic poplar, conferring resistance of the plants to *Xanthomonas populi* pv. *populi* [63]. AMP 3.1, designed by Kang et al. [64], has been tested only in clinical gram-negative pathogenic bacteria such as *P. aeruginosa* and *E. coli*. These promising results are also highlighted in Gomes et al. [65] for a broad range of skin infectious bacteria. The salivary peptide Dhvar-5 was shown to inhibit *Staphylococcus aureus* [66] and fungi such as *Aspergillus furmigatus* [67]. The mechanism of action of this peptide, rather than primarily involving membrane disruption, appears to be slow translocation across the membrane to reach the cytoplasm [68]. The CA-M peptide, also known as CA(1-7)M(2-9) [69], has shown success to control antibiotic-resistant bacteria [70]. Recently, a possible mechanism of functioning of this peptide conjugated with high temperatures has been hypothesized, leading to condensation of the phospholipidic bilayer and consequent membrane destabilization that leads to cell death without pore formation [71]. AMPs have also been highlighted for their synergic potential when mixed together [54,72,73]. Despite most of these AMPs having revealed antimicrobial activity to clinical bacterial isolates, their action towards phytopathogenic bacteria is unknown.

To investigate the potential of AMPs concerning antimicrobial activity, a set of techniques has been used to distinguish and appoint the more efficient peptides [74]. Minimal inhibitory and bactericidal concentrations, MIC and MBC, respectively, have been the most common strategy to evaluate AMPs [29,52,75]. Likewise, the analysis of bacterial growth is also widely used in investigating the peptides bioactivity [76]. To understand the function of the peptides in membrane disruption and cell viability rate, flow cytometry has been highlighted as a fast and reliable method [77,78].

This work was aimed at characterizing the activity of six AMPs (BP100, RW-BP100, CA-M, 3.3, D4E1, and Dhvar5); five of them never tested against Psa (RW-BP100, CA-M, 3.3, D4E1, and Dhvar5). The AMP broad action against 22 Psa strains isolated in Portugal and a Psa3 reference strain isolated in Italy has been assessed as a different approach to understand the range of bioactivity of the peptides in the same pathovar. The mechanism of action of these AMPs was investigated by flow cytometry. Additionally, the advantage of using mixtures of the peptides to improve the antimicrobial efficiency was assessed. Finally, the virulence capacity of the strains treated with peptides was inspected on tobacco cv. ‘Havana’ leaves with the hypersensitivity response (HR) test.

## 2. Results

### 2.1. Peptide Synthesis

All peptides listed in Table 3 were synthesized by standard solid-phase peptide synthesis (SPPS) protocols based on the Fmoc/tBu orthogonal protection scheme [79], in the Laboratory of Peptide and Peptide-Nucleic Acid Synthesis of the Faculty of Sciences of the University of Porto (POP-UP). The peptides were isolated with high purity (≥98%), according to reverse-phase high performance liquid chromatography (RP-HPLC) analysis, and their expected molecular weights (MW) were confirmed by electrospray ionization-ion trap mass spectrometry (ESI-IT MS) (Figures S1–S12 in Supplementary Materials).

### 2.2. Antibiogram Assays

**Figure 1:**
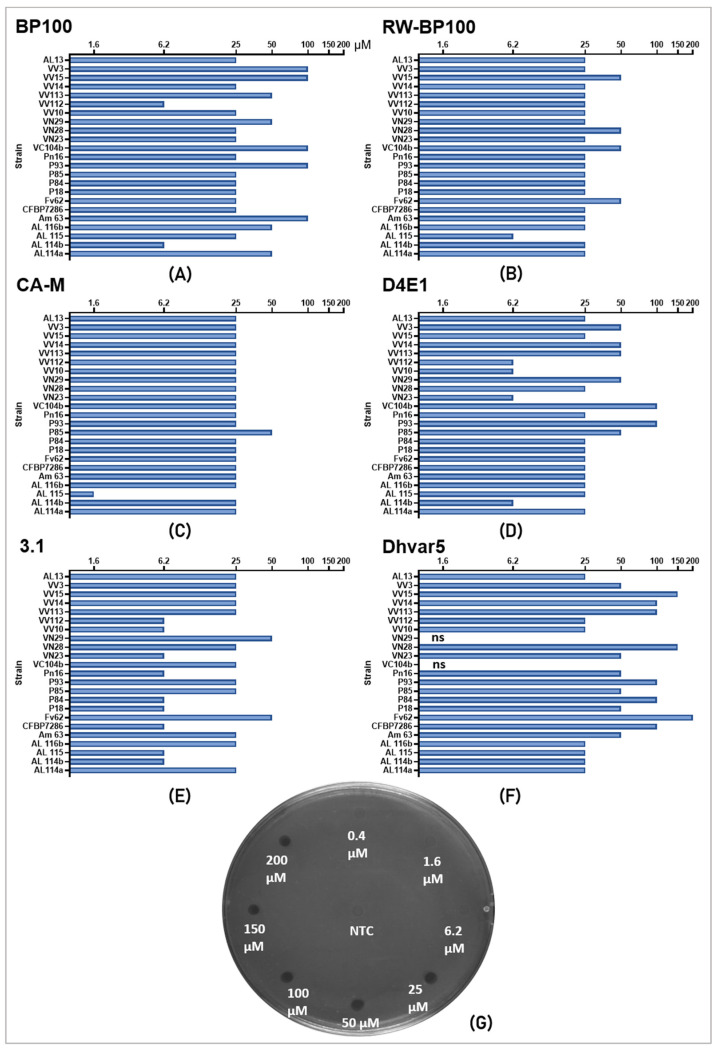
Antibiogram results of peptides (**A**) BP100, (**B**) RW-BP100, (**C**) CA-M, (**D**) D4E1, (**E**) 3.1, and (**F**) Dhvar-5 against Psa. “ns” means the strain was not susceptible to the peptide in the tested concentration range. All values correspond to concentrations in µM. (**G**) Represents the antibiogram assay to Psa carpet in plate, NTC means the negative control (H_2_O). The assay was repeated three times.

The antibacterial activity of the peptides BP100, RW-BP100, CA-M, D4E1, 3.1, and Dhvar-5 on Psa was first screened with antibiograms to compare in vitro the susceptibility of the Psa strains to each peptide (Figure 1). Overall, three peptides—RW-BP100, CA-M, and 3.1—exhibited higher antibacterial activity whatever the Psa strain assayed. RW-BP100 and 3.1 were mostly efficient at a range of 6.2 to 25 µM, despite some strains only being inhibited at 50 µM. For RW-BP100, strain AL115 showed high susceptibility at 6.2 µM, while four strains (Fv62, VC104b, VN28, and VV15) were less susceptible, with inhibition at 50 µM. Regarding the 3.1 peptide, nine Psa strains (VV112, VV10, VN23, Pn16, P84, P18, CFBP7286, AL115, and AL114b) showed high susceptibility at 6.2 µM, while two strains (Fv62 and VN29) were inhibited at 50 µM. CA-M displayed antibacterial activity mostly at 25 µM, with P85 being inhibited at 50 µM, and AL 115 showing higher susceptibility (inhibition at 1.6 µM). BP100 and D4E1 displayed a similar activity, i.e., antibiogram values ranged between 6.2 and 100 μM; however, some variability was observed among the strains. Dhvar-5 displayed the lowest activity, being efficient mostly at higher concentrations. Additionally, two strains (VN29 and VC104b) showed no susceptibility in the range of concentrations assayed. 

This evaluation indicated a distinct range of AMP inhibitory concentrations between 22 Psa strains. Thus, eight Psa strains were selected, according to higher and lower susceptibility in antibiogram analysis (CFBP7286, AL114b, AL115, Fv62, P85, VN29, VV10, and VV112), for further analysis as described in the next section.

### 2.3. Minimal Inhibitory and Bactericidal Concentration and IC_50_

The synthetic peptides RW-BP100, CA-M, and 3.1 were tested in vitro for their inhibitory and bactericidal activity against eight Psa strains (Table 1) according to the effective range of concentrations obtained in the antibiograms. BP100 was used as a reference once it had already been tested against Psa by Oliveras et al. [29].

BP100 showed a low MIC of 3.4 µM against all tested strains. The Psa strains AL114b, AL115, and CFBP7286 showed similar values for MBC and MIC. On the other hand, its bactericidal effectiveness in Psa strains is higher for P85, VN29, and VV10 (MBC at 6.2 µM), with the highest value observed for strains Fv62 and VV112 (MBCs at 10 µM) (Table 1). 

For most of the strains, the CA-M’ and BP100 bioactivity were identical (MIC at 3.4 µM), being higher for strains P85 and VN29 (MIC at 6.2 µM). Regarding MBC, both CA-M and RW-BP100 showed the same value for most of the strains (MBC at 6.2 µM) except for AL114 and FV62. The highest values of MICs and MBCs were obtained for peptide 3.1, regardless of the Psa strain studied (Table 1).

**Figure 2:**
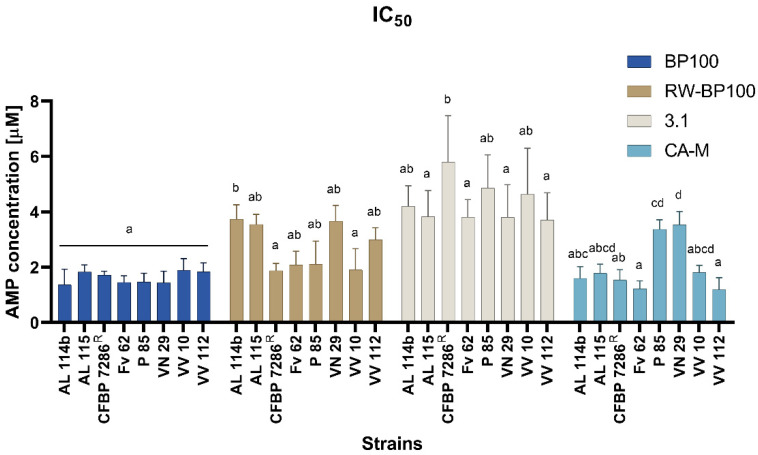
Half-inhibitory concentration (IC_50_) of eight Psa strains against four AMPs: BP110, RW-BP100, 3.1, and CA-M. The assay was repeated three times and analyzed in GraphPad^TM^ with one-way ANOVA. Letters in the top of the bars means significant differences for each AMP assay.

**Figure 3:**
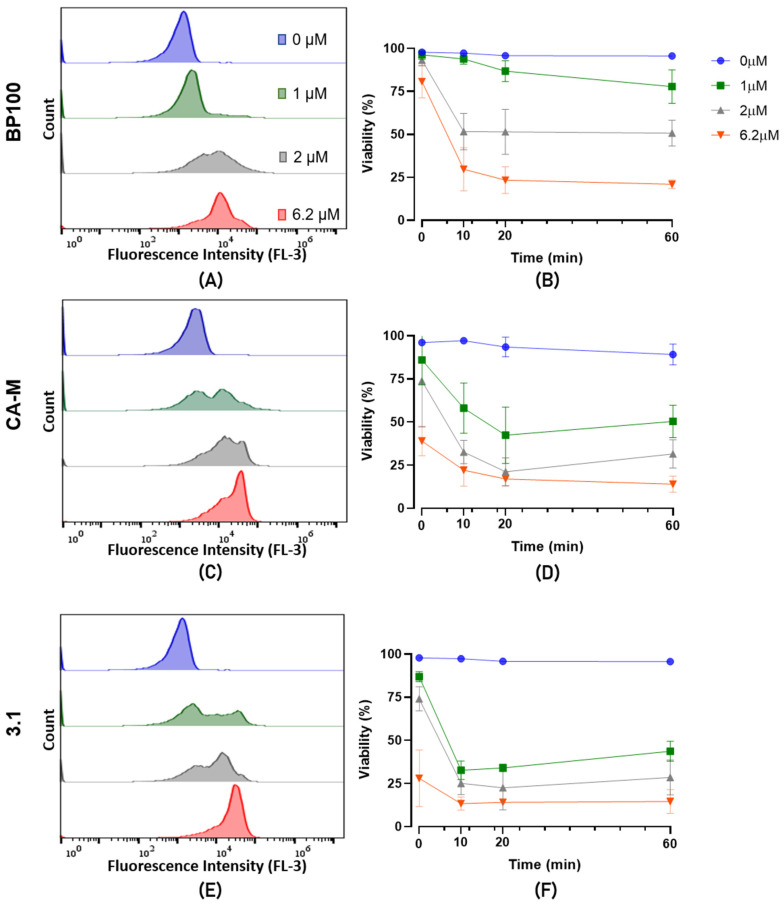
Flow cytometry analysis of PI-stained CFBP7286 Psa treated with (**A**,**B**) BP100, (**C**,**D**) CA-M, and (**E**,**F**) 3.1. Treatments consisted of 1 h with the corresponding peptide at (blue, ●) 0 µM, (green, ■) 1 µM, (grey, ▲) 2 µM, and (red, ▼) 6.2 µM. (**A**), (**C**), and (**E**) represent the fluorescence peak on FL3 detector showing the self-fluorescence at 0 µM to PI-detected cells shifting to the right with AMPs treatments. The assay was repeated three times.

The half-maximal inhibitory concentration (IC_50_) for these AMPs, based on bacterial growth curves (Figures S14–S17), revealed a similar concentration (~2 µM) for BP100 and CA-M, for all strains except for P85 and VN29, which showed a higher IC_50_ (~4 µM) for CA-M treatments (Figure 3). BP100 showed a more uniform response among the strains (*p* < 0.05) according to the IC_50_ (Figure 2). The 3.1 peptide requires higher concentrations to inhibit the growth of the eight Psa strains (Figure 2). RW-BP100 also demonstrated more variable results among the strains (Figure 2).

### 2.4. Evaluation of Bacterial Viability

The cell lysis of the Psa CFBP7286 treated with peptides BP100, CA-M, and 3.1 was assessed by flow cytometry. After 1 h of exposure, a shift towards higher fluorescence peaks was observed to the right of the histogram, which increases with the peptide concentration (Figure 3A–C). This increase of fluorescence suggests a higher interaction of the propidium iodide (PI) with the bacterial DNA, meaning that these peptides increase the permeability of the membrane or its disarrangement. In general, for these three peptides, the 6.2 µM concentration showed a higher number of PI-stained cells (Figure 3A–C).

The assessment of viability performed at t_0_, t_10_, t_20_, and t_60_ minutes (t means time), showing a decrease in cell viability (due to membrane disturbance or cell death) with the increase of the peptides’ concentration (Figure 3D–F).

The instantaneous effect of the peptides is seen at the t_0_ time point. CA-M and 3.1 peptides resulted in higher membrane permeabilization than BP100 (Figure 3A–C) at this time point. For these AMPs, 6.2 µM induced fluorescence between 50 to 75%, compared to around 20% of BP100 (Figure 3A–C). This higher efficiency in inducing instantaneous membrane permeabilization of CA-M and 3.1 was also observed for lower concentrations (1 µM). After 60 min 25% of Psa cells incubated with BP100 showed PI-fluorescence with a concentration of 1 µM, while in CA-M and 3.1, this value was over 50%. Interestingly, we verified a phenomenon of recovery of the Psa viability (from t_10_ and t_20_ to t_60_) at lower concentrations of 3.1 (Figure 3F). At 2 µM, the average of viable cells was also low for 3.1 and AMP and similar between BP100 and CA-M. At a higher concentration, the viability was reduced after 60 min to a similar level in all peptides. Isopropyl alcohol 23% was used as a positive control and showed high efficiency in permeabilizing the cells to PI from t_0_ to t_60_.

**Figure 4:**
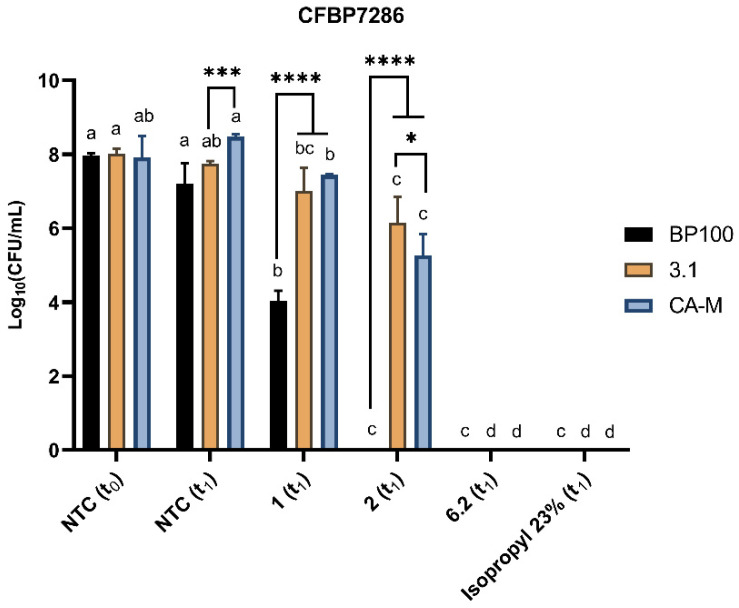
Evaluation of CFUs of CFBP7286 after 1 h (t_1_) treatment with BP100, CA-M, and 3.1 at 1, 2, and 6.2 µM in comparison to non-treated cells (NTC) at t_0_ and t_1_. Isopropyl alcohol at 23% is a positive control. Statistical letters showing differences (*p* < 0.05) throughout the treatment of each AMP treatment. *, ***, and **** refer to the statistical significances for differences in each time point of the analysis with *p* < 0.05, *p* < 0.001, and *p* < 0.0001, respectively. The assay was repeated three times and analyzed with a one-way ANOVA (GraphPad^TM^). Letters in the top of the bars means significant differences.

Colony forming units (CFUs) were counted to confirm the flow cytometry viability data. These assays were carried out with Psa cells exposed for 1 h (t_1_) to the AMP. At this time point, compared to the negative control, CFUs results suggest a decline of viable cells, except for the 3.1 where treated and not treated cells showed no significant differences (*p* < 0.05) to 1 µM treatment (Figure 4). A higher compromise of viability/cell division was observed in BP100 with a reduction of 50% of CFUs in cells treated with 1 µM (Figure 4). CA-M and 3.1 peptides presented a slight decrease of CFUs at 2 µM, and cell death was only recorded at 6.2 µM treatments.

**Figure 5:**
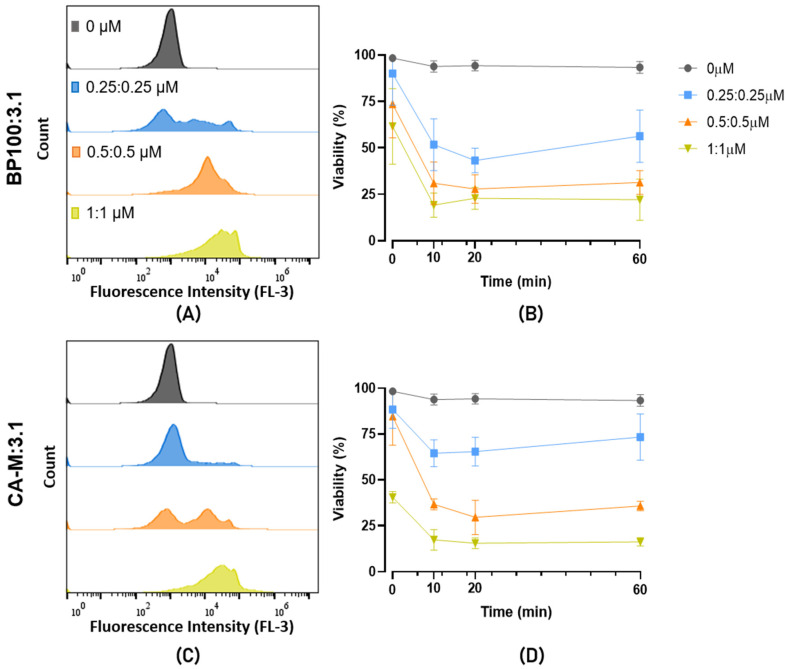
Flow cytometry analysis of PI-stained CFBP7286 Psa treated with peptide mixtures BP100:3.1 (**A**,**B**) and CA-M:3.1 (**C**,**D**). Treatments consisted of 60 min analysis being A and C the fluorescence peaks from FL3 detector at t_60_. Psa viability evaluation (%) is shown in (**B**) and (**D**), with four times of analysis, t_0_, t_10_, t_20_ and t_60_, with the corresponding peptide mixtures of (gray, ●) 0 µM, (blue, ■) 0.25:0.25 µM, (orange, ▲) 0.5:0.5 µM and (mustard, ▼) 1:1 µM. The assay was repeated three times.

Putative synergic effects of mixing the AMPs on cell viability were investigated by flow cytometry. The mixture of CA-M:3.1 showed a faster membrane destabilization (t_0_) than BP100:3.1 at high concentration (1:1 µM) (Figure 5). On the other hand, the low doses of both mixtures induced a slight decrease in cell viability with 25% for CA-M:3.1 and 40% for BP100:3.1. For both mixtures, the two higher concentrations reached a similar viability loss of ~75% (Figure 5B,D).

**Figure 6:**
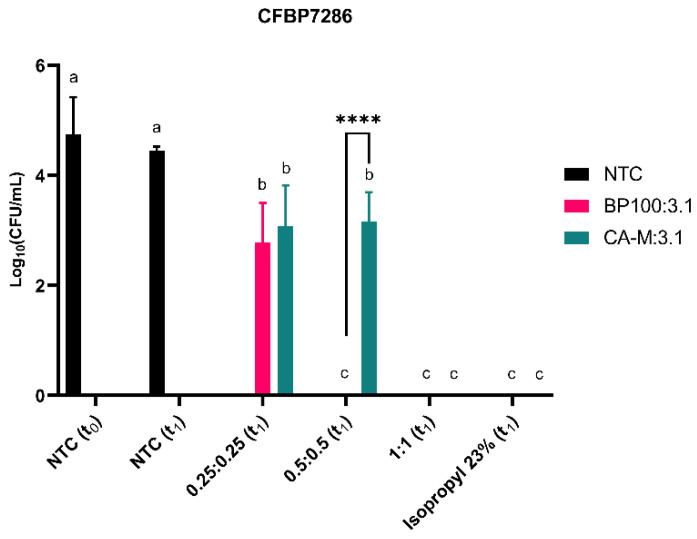
Evaluation of cell viability of CFBP7286 Psa at CFUs after 1 h of treatment (t_1_) with two AMPs mixtures: BP100:3.1 and CA-M:3.1. The combinations used were at 0.25:0:25, 0.5:0.5 and 1:1 µM for both mixtures. Non-treated cells (NTC) were used at t_0_ and t_1_. Isopropyl alcohol at 23% is a positive control. Statistical letters represent the differences between AMPs concentrations and NTC for each mixture. **** means a statistical significance with *p* < 0.0001. The assay was repeated three times and analyzed with a one-way ANOVA (GraphPad^TM^). Letters in the top of the bars means significant differences.

The efficiency of the AMPs mixtures on cell viability/division was assessed by CFUs analysis. The BP100:3.1 mixture was the most efficient against Psa with a decrease (*p* < 0.05) in the number of viable cells at low concentration and did not show recovered Psa cells from other higher concentrations and positive control (Figure 6). CA-M:3.1 was lethal only at the higher concentration, although a slight and similar decrease in cell viability was observed at the low doses tested (0.25:0.25 and 0.5:0.5 µM) (Figure 6).

### 2.5. Evaluation of AMPs Treatments on Tobacco Hypersensitivity Response

**Figure 7:**
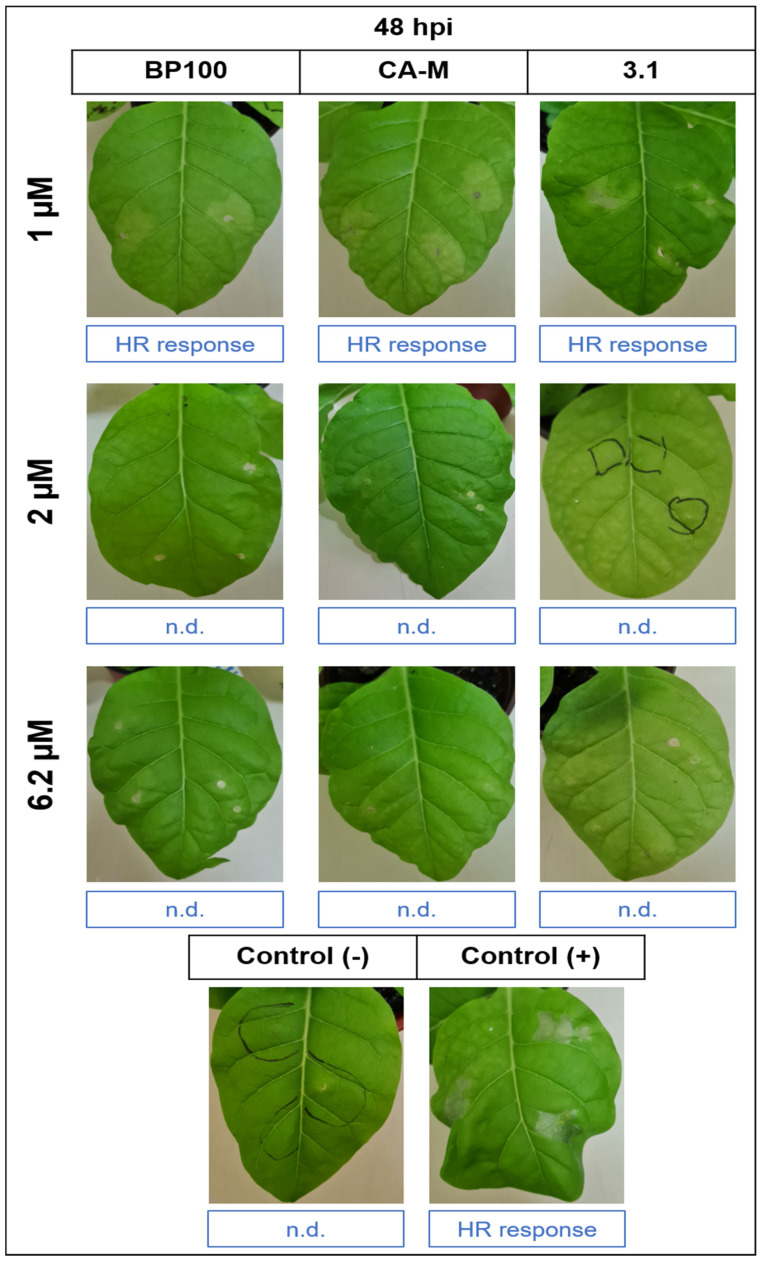
HR in *N. tabacum* leaves 48 h post-inoculation (hpi) of Psa CFBP7286 treated with individual peptides, namely BP100, CA-M and 3.1 in three different concentrations (1, 2, and 6.2 µM). HR response means the positive hypersensitivity response and n.d. means HR was not detected.

The HR bioassays in tobacco leaves provides information on the impact of AMP treatment on Psa virulence. All treatments at 1 µM showed an HR (Figure 7), with the spots being less intense than those verified in the positive control. In the rest of the concentrations for all AMPs, treated cells did not show an HR in tobacco, and only some burned halos appeared in the infiltration site, which can also be seen in the negative control (Figure 7). Results showed high symptomatic spots in *Nicotiana tabacum* leaves in the positive control treatment (+) under non-treated Psa CFBP7286 after t_1_ in the same conditions as the treated cells (Figure 7). The negative control (−) did not show any virulence symptoms. Controls were also made with the AMPs alone, leading to a small burn halo at the syringe tip contact during the infiltration. 

## 3. Discussion

The sustainable control of BCK urges to find out biomolecules with antimicrobial activity, of which bactericidal AMPs became a promising alternative [80,81]. AMPs’ mechanisms of action generally differs from those of conventional antibiotics to control pathogenic infections [43,45] and plant pests (Vestaron company, Kalamazoo, MI, USA [82]), thus representing a direction for a new post-antibiotics era. In the field of bacteria phytopathology, diverse AMPs have been tested against, e.g., *Xanthomonas* sp. [54], *Pseudomonas syringae* [56], and *E. amylovora* [48], revealing encouraging results. However, few studies have focused on finding new AMPs with high specificity against Psa [28,29,30]. This research represents a contribution to fill this gap, by investigating the antimicrobial capacity of six AMPs, five of which have never been tested against Psa, in comparison with the well-studied BP100. In fact, the bactericidal effect of BP100 on Psa has already been emphasized by Oliveras et al. [29]. Furthermore, of the other five AMPs, only D4E1 had been screened against phytopathogenic bacteria [53]. Beyond the novelty to address AMPs’ bioactivity by flow cytometry and using a PSA-virulence analysis (HR) against Psa, this study evaluates the efficiency of each AMP on an assortment of field-collected Psa isolates, and against the Psa reference strain CFBP7286. Additionally, we assayed mixtures of BP100, CA-M, and 3.1, aiming to further elucidate the recent findings pointing towards a synergistic effect of AMPs, particularly if presenting different mechanisms of action, which might increase their potential application in a real case [54,72].

Antibiograms have been routinely used to address AMPs bioactivity against different pathogenic bacteria [83,84]. In this study, the antibiograms showed a different susceptibility of the 22 Psa strains to the six AMPs assayed, and allowed to identify a bacteriostatic efficiency gradient for the different AMPs with RW-BP100 = CA-M > BP100 > 3.1 > D4E1 > Dhvar5. Despite the lower activity of Dhvar-5, this AMP has been described as having the capacity of crossing the membrane at a slow rate [68], which, thus, may delay its antimicrobial activity. Due to its alleged different mechanism of action, this AMP deserves further molecular studies to analyze its real antimicrobial efficiency against Psa, alone or mixed with other AMPs. MIC assays have been performed for some of these AMP in several phytopathogenic bacteria [49,54,76]. In the current study, BP100 and CA-M showed the same MIC value of 3.4 µM for most of Psa strains with CA-M ranging up to 6.2 µM in two strains. When comparing the minimal concentration of AMPs required for Psa growth inhibition and cell death, the data showed that MBC values were higher than MICs for most of the AMPs and the strains studied, suggesting that within the MICs–MBC range of AMPs concentrations growth inhibition was not irreversible, as has been shown in other studies. For example, Velivelli et al. [52] demonstrated that MtDef5 AMP had an MIC value between 6 and 12 µM for *X. campestris,* and an MBC value of 12 µM. Additionally, *P. aeruginosa*, a clinic pathogen, showed different values of MIC and MBC in response to AMPs [85].

Several peptides were tested against phytopathogenic bacteria, showing MIC ranges of 6.2–50 µM for *E. amylovora*, *P. syringae* pv. *syringae*, *X. fastidiosa*, and *X. axonopodis* pv. *vesicatoria* and of 0.8–50 µM for *X. arboricola* pv. *pruni.* In Psa, MICs ranged between 1.6-50 µM, with a low MIC value for KSLW, while other AMPs had values above 12.5 µM [30]. Besides these results, BP100 and BP100-analogs were also tested against Psa, revealing an MIC range between 1.6 to 25 µM, having BP100 higher efficiency, with an MIC value of 1.6 µM [29]. However, in this example, the authors used a single and local strain, e.g., Psa3700.1.1 [29], thus assessing the AMPs efficiency across different Psa strains, important to disclose the behavior of distinct Psa population to BP100. The BP100 analog RW-BP100 was previously reported to have a better higher bacteriostatic (MIC) and bactericidal (MBC) activity in gram-negative bacteria *E. coli* and P. aeruginosa than BP100 [60]. Regarding the current results, the Psa susceptibility was higher to BP100 (3.4 µM) than to RW-BP100 (6.2 µM), except in the reference strain that had the same susceptibility for both peptides. Concerning other AMPs, the promising inhibitory concentrations of CA-M against a multi-drug resistant *Staphylococcus aureus* strain, ranged between 0.22–0.44 µM, which highlights the potential of this AMP [70]. Although such low MIC values were not verified for the Psa strains tested in the current work, its antimicrobial efficiency (most MIC values at 3.4 and MBC at 6.2 µM) was close to that of BP100, which is encouraging as an alternative to BP100 to control Psa. These results are also supported by Oliveras et al. [29] and Camó et al. [34] AMPs surveys for Psa control. AMP 3.1 has shown activity on gram-negative bacteria, such as *E. coli*, with an MIC value around 6 µM [64,65], while showing a broader range in *P. aeruginosa*, namely from 3 [65] to 25 µM [64]. Compared with these data, 3.1 needed higher concentrations to have bacteriostatic activity in Psa. Nevertheless, these results are in line with other reports of AMP 3.1 activity on other gram-negative bacteria. The IC_50_ values are according to inhibitory concentrations to Psa, with slight variations depending on the target strain. The MIC, MBC, and IC_50_ of Fv62 strain reveal his Psa strain low susceptibility to all AMPs tested. Based on the main function of most of these AMPs as bacterial membrane destabilizers, these results may indicate differences among the strains’ membrane composition. Thus, it evidences the potential of further analyses to investigate the Psa board range considering the diversity of strains/populations from the same species or pathovar in future investigations for efficient AMPs.

Membrane disorders induced by AMPs can be assessed by flow cytometry [77,78]. Thus, CA-M and 3.1 demonstrated fast membrane destabilization at t_0_ (Figure 3). Additionally, these two peptides had a lower critical concentration to destabilize cell membranes at 1 µM than BP100. This fact revealed the potential of these peptides to be supporting actors in combination with each other or with other molecules once they change the membrane stability and might contribute to the input across the membrane. At low concentrations (1 µM and 2 µM), these two AMPs have reduced bactericidal efficiency, which suggests that despite inducing a fast membrane destabilization (allowing some of the fluorescence DNA marker—PI—to internalize), their effects may not be too severe, allowing Psa cells to recover. At higher concentrations, both AMPs promoted higher disorder in the membrane, increasing the bactericidal efficacy (Figure 4). The effects of BP100 on membrane destabilization by flow cytometry were previously reported in the phytopathogenic bacteria *E. amylovora* [48]. According to the different roles and efficiency revealed by these AMPs against Psa, the possibility of a synergic effect on bactericidal performance is apparent. The synergic effect of different AMPs has been described as a promising strategy to control pathogenic bacteria [71,72,73]. Topman et al. [54] demonstrated that mixtures of AMPs (FK-20 and FdK-20) improved the bacteriostatic and bactericidal effects, reducing in vitro the growth of *Xanthomonas* sp. and *P. syringae* pv. *tomato* and decreasing the host-symptoms in planta assays. The use of the two mixtures of AMPs against Psa showed the high efficiency of the BP100:3.1 combination, with fast membrane destabilization associated with a strong bactericidal efficiency (Figure 6). Although the CA-M:3.1 mixture also promoted rapid membrane damage (Figure 5), its bactericidal efficiency was lower than that of BP100:3.1. The approach of a better bactericidal AMP, i.e., BP100 (Figure 3 and Figure 4), with a faster membrane destabilizer AMP, such as 3.1 (Figure 3 and Figure 4), improves the peptide efficiency against Psa. The lethal efficiency of BP100 was increased from a concentration of 2 µM (Figure 4) to 0.5 µM (Figure 6), when combined with 3.1 AMP at equal concentrations. Synergic activity at low concentrations of AMPs reveals a breakthrough in the search of the AMPs with strong antimicrobial activity on Psa. 

The efficacy of AMPs in reducing the virulence of AMP-treated strains was assessed by comparing HR symptoms (Figure 7). These vary with the AMP combinations/concentrations and may be related to a reduction of the bacterial density, as demonstrated in the CFUs of the treated strains (Figure 4). Despite the CFUs of both live cells treated with and 1 µM and 2 µM being similar (*p* < 0.05), only those from 1 µM treatment induced an HR (Figure 4 and Figure 7). The increase of 3.1 concentration, at non-lethal doses, might, however, interfere with the quorum sensing and/or bacterial virulence involved in inducing an HR in tobacco leaves. As reviewed by Duperthuy [86], AMPs at sub-inhibitory and sub-lethal concentrations can modulate the virulence in gram-negative bacteria. The AMPs at these concentrations have been reported to downregulate bacterial virulence through sub-cellular interaction [87], thus deregulating the gene expression involved in virulence and bacteria resistance to environmental stress [86]. These major effects of AMPs’ action at sub-inhibition growth levels have also been hypothesized to be related to quorum sensing, virulence, and biofilm formation. Recent data suggest that quorum sensing might be affected by AMPs, consequently decreasing the virulence and biofilm production [88].

The studies of the bioactivity of the AMPs per se on plants’ performance is a pre-requisite to ensure no toxicity, and their potential use for control strategies. Thus, AMP toxicity was also verified on injury tissues by syringe infiltration on tobacco plants. The tested AMPs demonstrated an absence/low toxicity mostly evidenced by BP100, CA-M, and 3.1, showing only slightly burned spots, mostly resulting from the syringe tip adapter contact in the infiltration process (Figure 7, Figure S13). The assays on tobacco plants are often used to analyze the toxicity effects of AMPs [29,30,49,57]. Camó et al. [30] assessed the toxicity of a broad spectrum of peptides, also showing a low level of toxicity, in line with our results. The same approach was applied to several AMPs (Oliveras et al. [29]), which were found to induce small damages, with smaller lesion-spots than those of the positive control (with <20 mm diameter)). These effects increased with peptides concentration (12.5 mm to 250 µM), which supports our data, including for the BP100, which caused tobacco leaf lesions of 4 mm at 50 µM and or 12.5 mm at 250 µM [29].

This work contributes to disclose new AMPs that, alone or in mixtures, have potential towards the control of Psa. This efficacy can, thus, be used as a complement to the currently available methods for containment of bacterial phytopathogens. Furthermore, the demonstrated added value of the AMPs mixtures can be explored for future industrial applications helping the kiwifruit producer to control BCK. In particular, the fast capacity of 3.1 peptide to destabilize membranes at low concentrations, probably by inducing pore formation, increases its potential to be used as a therapeutic facilitator synergically with other AMPs or different molecules in Psa treatments. In other words, this study suggests that the synergic action of different AMPs should be better explored. These results indicate possible future perspectives on the redesign of 3.1 and BP100 AMPs to increase their activities, and to explore the use of these peptides to produce transgenic plants.

## 4. Materials and Methods

### 4.1. Bacterial Strains and Growth Conditions

Psa bacterial strains used in this study are listed in Table 1. All strains were obtained from stocks preserved at –80 °C and cultured in King’s B medium (protease peptone, 20.0 g; anhydrous K_2_HPO_4_, 1.5 g; glycerol, 10.0 mL; MgSO_4_·7H_2_O, 1.5 g; agar, 1.2 g; distilled water up to 1.0 L). Individual colonies of each strain were selected and re-streaked two times to ensure purity. Psa strains were isolated in different infected kiwifruit orchards between 2013 and 2017. CFBP7286 was used as Psa biovar 3 reference strain. For the antibiogram assays, pure colonies of all bacterial strains were grown overnight in Muller-Hinton Broth (MHB; Oxoid), at 28 °C and 240 rpm. Bacterial growth was monitored and adjusted to 0.135 optical density at 600 nm (OD_600_) in a spectrophotometer (Genesys 10uv Scanning, Thermo Scientific). For the MIC and MBC assays, eight Psa strains (CFBP7286, AL114b, AL115, Fv62, P85, VN29, VV10, and VV112) were cultured on MHB medium and grown overnight at 25 °C and 200 rpm and bacterial concentration was diluted in MHB to OD_600_ 0.1. For flow cytometry experiments, bacteria were cultured in King’s B broth medium for 20 h, at 25 °C in a shaking incubator at 200 rpm.

### 4.2. Peptide Synthesis

AMPs synthetized in this study, respective amino acid sequences and properties are listed in Table 2. Peptides were assembled by SPPS on an automated Symphony X synthesizer from Gyros Protein Technologies (Tucson, AZ, United States), through the orthogonal protection scheme Fmoc/tBu [79], using a Rink Amide MBHA LL resin (100–200 mesh, 0.33 mmol/g, NovaBiochem) pre-conditioned in dimethylformamide (DMF, Merck, Darmstadt, Germany) for 10 min. Then, the Fmoc protecting group was removed by treating the resin twice with a solution of 20% piperidine (Sigma-Aldrich, St. Louis, MO, USA) in DMF for 5 min. The relevant *N*^α^-Fmoc-protected amino acid (Fmoc-AA-OH) was next coupled to the deprotected resin, followed by treatment with a solution containing 100 mM of Fmoc-AA-OH, 200 mM *N*-methylmorpholine (NMM), and 100 mM of the in situ coupling reagent *O*-(6-chlorobenzotriazol-1-yl)-*N,N,N′,N′*-tetramethyluronium hexafluorophosphate (HCTU, NovaBiochem) in DMF. To deblock the α-amine group of the amino acid (AA) thus incorporated, for the ensuing coupling of the next Fmoc-AA-OH, the Fmoc protecting group was removed as previously described. The peptide chain was grown in the *C*- to *N*-terminal direction, alternating between coupling and deprotection cycles.

Once each peptide chain was fully assembled, it was cleaved from the resin, through a 2 h acidolytic cleavage, at room temperature and after shaking, using a trifluoroacetic acid (TFA)-based cocktail prepared in a ratio of 1 mL of cocktail for each 100 mg of resin with peptide. For cleavage of all peptides but RW-BP100, the cocktail was composed of 95% TFA (Merck), 2.5% triisopropylsilane (TIS, Sigma-Aldrich), and 2.5% deionized water. For peptide RW-BP100, having a high number of arginine residues, the cleavage cocktail consisted of 87.5% TFA, 5% deionized water, 5% phenol, and 2.5% TIS. 

Once the cleavage ended, the mixture was filtered through a sintered Hirsch funnel at reduced pressure, to collect the filtrate containing the crude peptide. The filtrate was diluted in 50 mL of methyl *tert*-butyl ether (MTBE, Sigma-Aldrich), and the solution transferred into a separatory funnel. Twenty-five mL of deionized water was added to the funnel, and liquid-liquid extraction was carried out, collecting the aqueous layer in an Erlenmeyer flask, and keeping the ethereal layer in the funnel. Another 25 mL of deionized water was added to the funnel, and the process was repeated, collecting the aqueous layer in the same Erlenmeyer flask. This procedure was repeated three more times, using 25 mL of deionized water in each of them. Finally, the organic layer was discarded and the pooled aqueous phase, containing the peptide, was freeze dried on a VirTis BenchTop Pro Freeze Dryer from SP Scientific (Warminster, PA, USA) and stored at −20 °C until subsequent purification.

Peptide purification was achieved by preparative scale RP-HPLC, on a Hitachi-Merck LaPrep Sigma system (VWR) equipped with an LP3104 UV detector and an LP1200 pump, using a reverse-phase C18 column (250 × 25 mm ID and 5 μm pore size, Merck). To confirm peptide purity, analytical RP-HPLC analyzes were performed on a Hitachi-Merck LaChrom Elite system equipped with a quaternary pump, a thermostated automated sampler and a diode array detector (DAD), using a reverse-phase C18 column (150 × 4.6 mm ID and 5 µm pore size, Merck); a mobile phase gradient from 1 to 100% acetonitrile (ACN, Merck) in 0.05% aqueous TFA was applied at a 1 mL/min flow rate, for 30 min, with detection at 220 nm. Peptide identity was confirmed by ESI-IT MS, using the direct injection mode of methanolic (LC-MS grade) solutions of the peptide. Finally, the purified peptide was freeze-dried and stored at −20 °C until further use.

### 4.3. Antibacterial Activity

#### 4.3.1. Antibiogram Assay

All six AMPs, BP100, RW-BP100, CA-M, D4E1, 3.1, and Dhvar-5 (Table 3), were evaluated by antibiogram assays to determine the most promising peptides against the selected bacterial strains. After lyophilization, peptides were solubilized in sterile distilled water, and working solutions of 0.4, 1.6, 6.2, 25, 50, 100, 150, and 200 µM were prepared. The concentrations used in this study were based on the similar studies looking for AMPs with potential against phytopathogenic bacteria [29,30]. 

For the pre-incubation, bacterial cultures were inoculated in Muller-Hinton (MH; Oxoid) agar (1.5%) medium by spreading 1 mL of each culture, removing the excess after 1 min and allowing drying for 15 min. One µL of each peptide dilution was loaded on previously inoculated MH agar plates and 1 µL of sterile distilled water was used as a negative control (Figure 1G). Plates were incubated at 25 °C for 24 h. After the incubation period, plate images were acquired on Gel Doc (BioRad, Hercules, CA, USA) and AMP activity was determined by no growth on drop site for each concentration. This experiment was repeated three times independently from different pure fresh colonies. 

#### 4.3.2. Determination of Minimal Inhibitory Concentration and Minimal Bactericidal Concentration of AMPs

The MIC and MBC were determined for peptides discriminated by the antibiograms as the most efficient, i.e., RW-BP100, CA-M, 3.1, and BP100. For this purpose, the Psa reference strain CFBP7286 was used as well as the strains AL114b, AL115, Fv62, P85, VN29, VV10, and VV112, which among the 22 Psa strains demonstrated a representative susceptibility for selected AMPs. In this assay, additional concentrations to those used in the antibiogram analysis were included in order to obtain more accurate results. Sterile 96-well microplates were filled with 150 µL of peptide and bacterial solution in a 1:1 ratio, leading to final concentrations of peptides equal to 0.25, 0.75, 1.6, 3.4, 6.2, 10, 25, 50, and 100 µM. As negative control a bacterial-free solution and sterile distilled water was used, and positive control with chlortetracycline to a final concentration of 25 µM was used. Microplates were incubated at 25 °C and 130 rpm and OD_600_ was measured hourly for 24 h, with 30 s vigorous shaking before each reading. The experiment was repeated three distinct times with different fresh bacterial colonies.

Data from bacterial growth curves were analyzed to determine IC_50_ values with two-way ANOVA and Tukey tests (*p* < 0.05). MIC values were set as the peptide lowest concentrations that reduced bacterial growth. MBC was defined as the lowest concentration that prevented bacterial growth after subculture on peptide-free King’s B agar plates, with further incubation at 25 °C, for 24 h.

#### 4.3.3. Flow Cytometry in the Evaluation of AMP Function and Viability

BP100 was selected for this assay in detriment of its analog RW-BP100 by better efficiency according to MIC analyzes. Additionally, CA-M showed similar MIC and MBC values to BP100 demonstrating as better candidates for future analysis. The 3.1 peptide, besides having proven bactericidal activity, was chosen for its potential revealed in the antibiogram analyzes, and its synergic capacity in AMP mixtures was investigated.

A flow cytometry method adapted from Benincasa et al. [89] was used to evaluate and quantify the loss of membrane integrity caused by the peptides BP100, CA-M, and 3.1 by discriminating between intact and damaged bacteria. Cultures of CFBP 7286 were grown overnight and then centrifuged at 2500 rpm for 5 min to harvest the cells. The supernatant was removed and the pellet was resuspended in sterile phosphate buffer saline (PBS; NaCl, 8 g; KCl, 0.2 g; Na_2_HPO_4_·12H_2_O, 2.9 g; KH_2_PO_4_, 0.2 g; distilled water up to 1.0 L; pH 7.0) to a final OD_600_ equal to 0.1. Bacteria suspension was mixed with the peptide with 1, 2, and 6.2 μM as the final concentrations in 24-well plates and incubated at 25 °C with 130 rpm. Propidium iodide (PI) with 10 μg/mL as the final concentration was added to each sample 20 sec before each reading time. The bacterial viability and PI uptake were assessed at 0 min, 10 min, 20 min, and 60 min. Since PI is a red fluorochrome not permeant to live cells and binds to DNA, it was used to detect cell lysis in a population [90]. Data were acquired with BD Accuri^TM^ C6 Plus Software, version 1.0.23.1, build 20151211.23.1 (Copyright © 2011 Accuri^®^ Cytometers, Inc., CA, USA) and analyzed with FlowJo (Becton Dickinson & Company, Sparks, MD, USA) and with GraphPad Prism 8 software. For each sample, 20,000 events were recorded. Untreated bacterial suspensions and 23% isopropyl alcohol-treated cells served, respectively, as negative and positive control. This experiment was independently repeated three times. 

#### 4.3.4. Assessment of Colony Forming Units (CFUs)

The number of viable bacteria cells of CFBP7286 strains was determined by CFU counting, using bacteria inocula exposed to the same AMPs (doses and periods of exposure) than those used on the flow cytometry assays. In the individual evaluation of BP100, CA-M, and 3.1 AMPs as well as the mixtures BP100:3.1 and CA-M:3.1, CFUs were determined by the drop method in the plate. All treated samples and controls were subjected to 10 serial ^1^/_10_ dilutions in sterile PBS and 10 μL of each dilution was cultured on King’s B agar (1.5%) plates, with further incubation at 25 °C for 24 h. After the incubation time, plates were recorded in a GelDoc (BioRad) and colonies were counted for the last dilution with bacterial growth. This experiment was performed in three biological and temporal replicates.

### 4.4. Hypersensitivity Response (HR) in Tobacco Leaves

To assess the HR, the Psa reference CFBP7286 strain was used. CFBP7286 cells were more exposed to the AMPs (doses and periods of exposure) than those used on the flow cytometry and the CFUs analyzes; from individual effects of AMPs on the reference CFBP7286 strain, the HR was assessed. Tobacco plants (*Nicotiana tabacum* cv. ‘Havana’) were sown in plant pots of 1 L in peat substrate (Siro, Mira, Portugal). Plants were grown for 8 weeks in a growth chamber with a controlled temperature at 25 °C ± 2 and relative humidity at 50% ± 5. Psa infiltration on tobacco plants for HR was performed according to Stefani and Loreti [91] using a syringe tip to inoculate 1 mL of bacterial suspension after 1 h of AMPs treatments in the abaxial side of the leaves. PBS was used as a negative control and Psa CFBP7286 at the same concentration and diluted in PBS for 1 h as the treatment was infiltrated as a positive control for HR. The AMPs at the assay concentration were prepared in PBS and infiltrated, also as a negative control of the treatment. The development of HR was monitored within 24 and 48 h post infiltration (hpi). Three tobacco plants, and three leaves of each plant, were used in three independent experiments. 

### 4.5. Statistical Analysis 

Statistical analysis was performed using GraphPad Prism software version 8.3.0. Multiple comparisons between the time-dependent effect of different concentrations of peptides were made using Two-way ANOVA test with Tukey’s test.

## Figures and Tables

**Table 1:** Comparison of MIC and MBC of the peptides BP100, RW-BP100, CA-M, and 3.1 against Psa strains. All values correspond to concentrations in µM. R means reference strain.

Strains	BP100		RW-BP100		CA-M		3.1	
	MIC	MBC	MIC	MBC	MIC	MBC	MIC	MBC
AL114b	3.4	3.4	6.2	6.2	3.4	10	25	25
AL115	3.4	3.4	6.2	6.2	3.4	6.2	10	10
CFBP7286 ^R^	3.4	3.4	3.4	6.2	3.4	6.2	10	25
Fv62	3.4	10	6.2	6.2	3.4	10	10	25
P85	3.4	6.2	6.2	6.2	6.2	6.2	25	25
VN29	3.4	6.2	6.2	6.2	6.2	6.2	10	25
VV10	3.4	6.2	6.2	6.2	3.4	6.2	10	25
VV112	3.4	10	6.2	6.2	3.4	6.2	10	25

**Table 2:** List of bacterial Psa strains used in biological assays.

Bacterial Strains	Host Plant	Origin	Year of Isolation
Al114a	*Actinidia deliciosa*	Amares, Portugal	2014
AL114b	*A. deliciosa*	Amares, Portugal	2014
AL115	*A. deliciosa*	Amares, Portugal	2014
AL116b	*A. deliciosa*	Amares, Portugal	2014
AL13	*A. deliciosa*	Amares, Portugal	2013
Am63	*A. deliciosa*	Amarante, Portugal	2013
Fv62	*A. deliciosa*	Felgueiras, Portugal	2013
P18	*A. deliciosa*	Vila de Prado, Portugal	2013
P84	*A. deliciosa*	Vila de Prado, Portugal	2013
P85	*A. deliciosa*	Vila de Prado, Portugal	2013
P93	*A. deliciosa*	Vila de Prado, Portugal	2013
Pn16	*A. deliciosa*	Penafiel, Portugal	2013
VC104b	*A. deliciosa*	Vila do Conde, Portugal	2013
VN23	*A. deliciosa*	Valença, Portugal	2016
VN28	*A. deliciosa*	Valença, Portugal	2017
VN29	*A. deliciosa*	Valença, Portugal	2017
VV3	*A. deliciosa*	Valença, Portugal	2016
VV10	*A. deliciosa*	Valença, Portugal	2017
VV14	*A. deliciosa*	Valença, Portugal	2017
VV15	*A. deliciosa*	Valença, Portugal	2017
VV112	*A. deliciosa*	Vila Verde, Portugal	2014
VV113	*A. deliciosa*	Vila Verde, Portugal	2014
CFBP7286 ^R, a^	*A. chinensis*	Latina, Italy	2008

^R^ Psa reference strain. ^a^ CFBP: Collection Française de Bactéries Phytopathogènes.

**Table 3:** Sequence and properties of peptides synthesized by SPPS.

Peptide	Sequence	Net Charge ^a,b^	MW (Da) ^b^
BP100	KKLFKKILKYL-NH_2_	6	1419.9
RW-BP100	RRLFRRILRWL-NH_2_	6	1583.0
CA-M	KWKLFKKIGAVLKVL-NH_2_	6	1769.2
D4E1	FKLRAKIKVRLRAKIKL-NH_2_	9	2079.4
3.1	KKLLKWLLKLL-NH_2_	5	1393.9
Dhvar-5	LLLFLLKKRKKRKY-NH_2_	8	1845.3

^a^ Estimated net charge at pH 7; ^b^ MW: molecular weight. Source: Pepdraw.com.

## Data Availability

Following the MDPI Research Data Policies, data from this paper will be available under request.

## Supplementary Materials

The following are available online. RP-HPLC chromatograms and ESI-IT MS spectra of test peptides.

## Sample Availability

Samples of the compounds are available from the authors to request in POP-UP (se.popup@fc.up.pt), University of Porto.
