# Supplementary material for: A Synergic Potential of Antimicrobial Peptides against Pseudomonas syringae pv. actinidiae
Source: Molecules. 2021 Mar 8;26(5):1461. doi: 10.3390/molecules26051461 (PMC7962642; doi:10.3390/molecules26051461)
Supplement: Supplementary file 1 [file molecules-26-01461-s001.pdf]

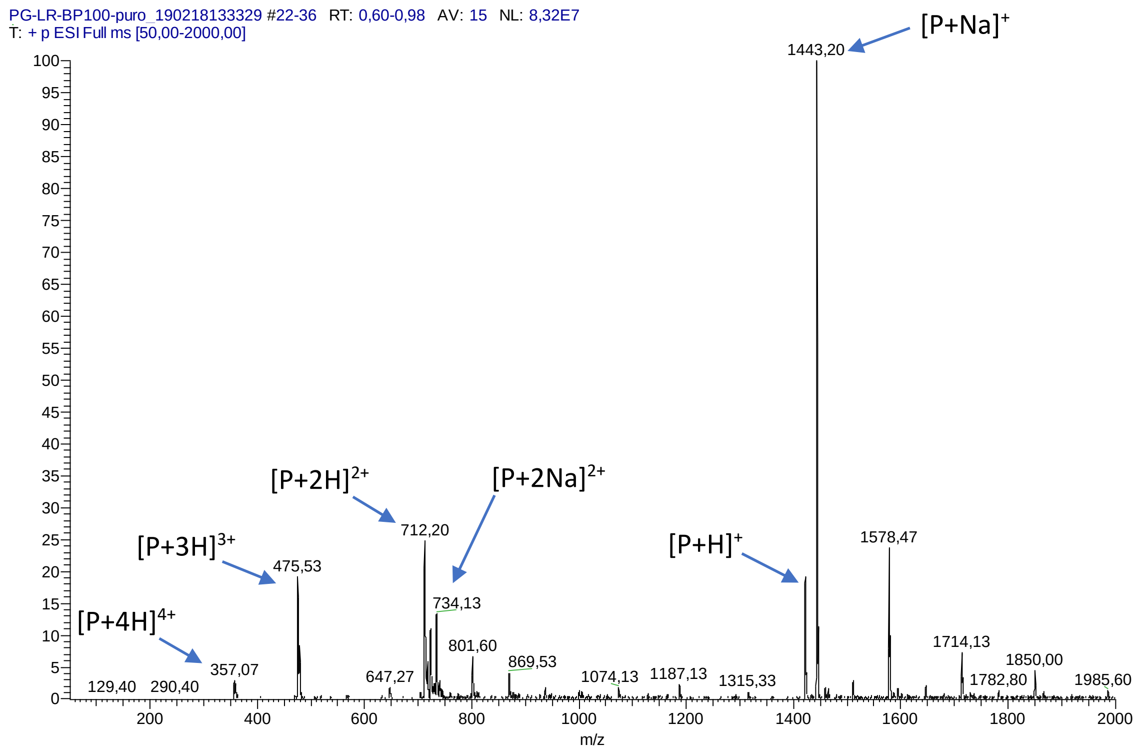

Figure S1. Mass spectrum (ESI-IT, positive mode) of peptide BP100 (MW=1419.9 Da), highlighting the quasi-molecular ion ($[P+H]^+$), its sodium adduct ($[P+Na]^+$, base peak), the di-protonated ($[P+2H]^{2+}$) ion and its sodium adduct ($[P+2Na]^{2+}$), and the tri-protonated ($[P+3H]^{3+}$) and tetra-protonated ($[P+4H]^{4+}$) ions of the target peptide (P).

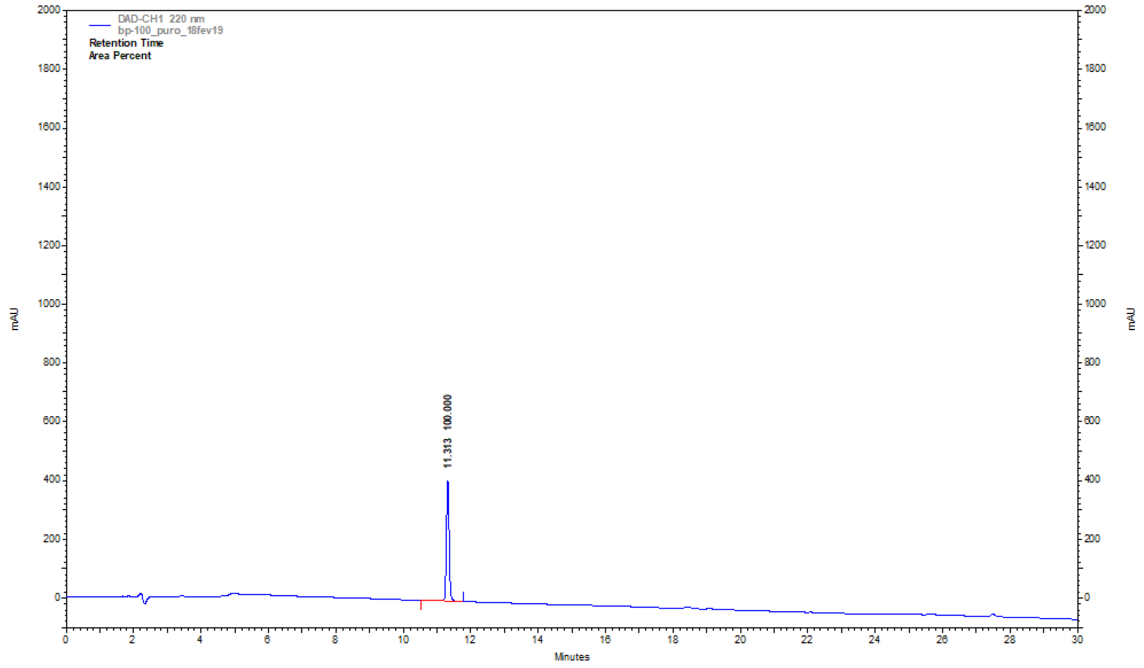

Figure S2. RP-HPLC chromatogram for peptide BP100, after purification; gradient elution from 1 to 100% ACN in 0.05% aqueous TFA at 1 mL/min flow rate, for 30 min, on a C-18 column (150 × 4.6 mm ID and 5 µm pore size); detection at 220 nm.

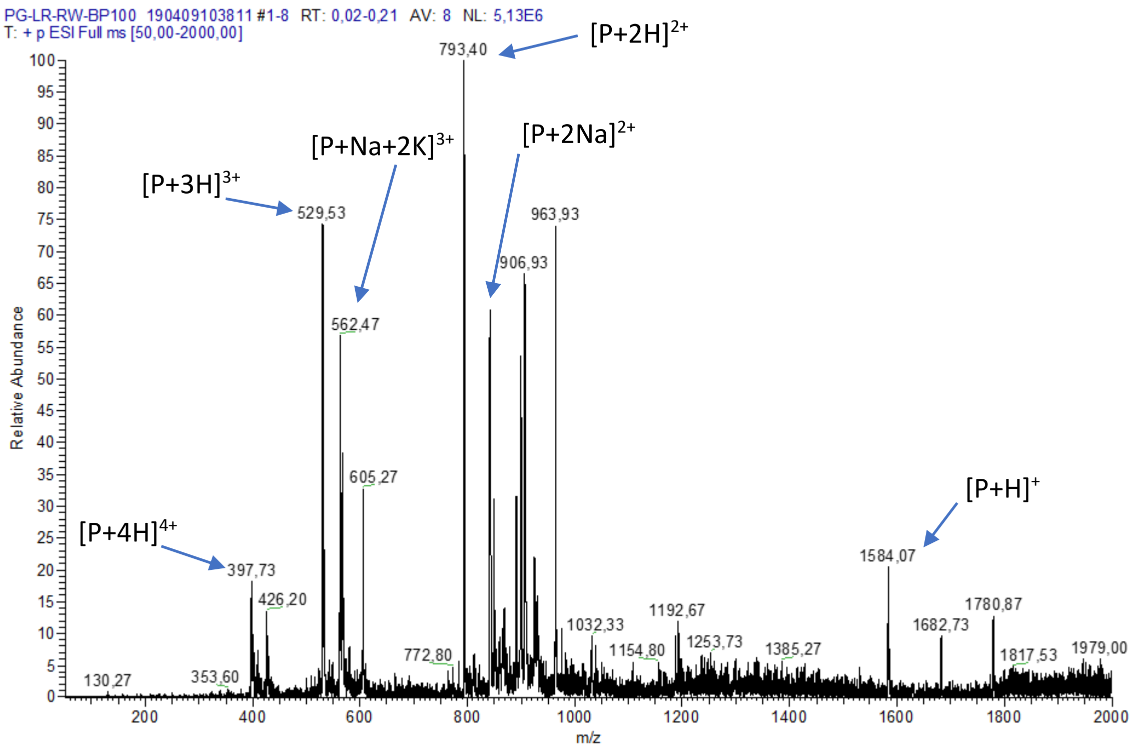

Figure S3. Mass spectrum (ESI-IT, positive mode) of peptide RW-BP100 (MW=1583.0 Da), highlighting the quasi-molecular ion ([P+H]$^{+}$), the di-protonated ([P+2H]$^{2+}$, base peak) ion and respective sodium adduct ([P+2Na]$^{2+}$), the tri-protonated ion ([P+3H]$^{3+}$) and its sodium and dipotassium adduct ([P+Na+2K]$^{3+}$), and the tetra-protonated ([P+4H]$^{4+}$) ion of the target peptide (P).

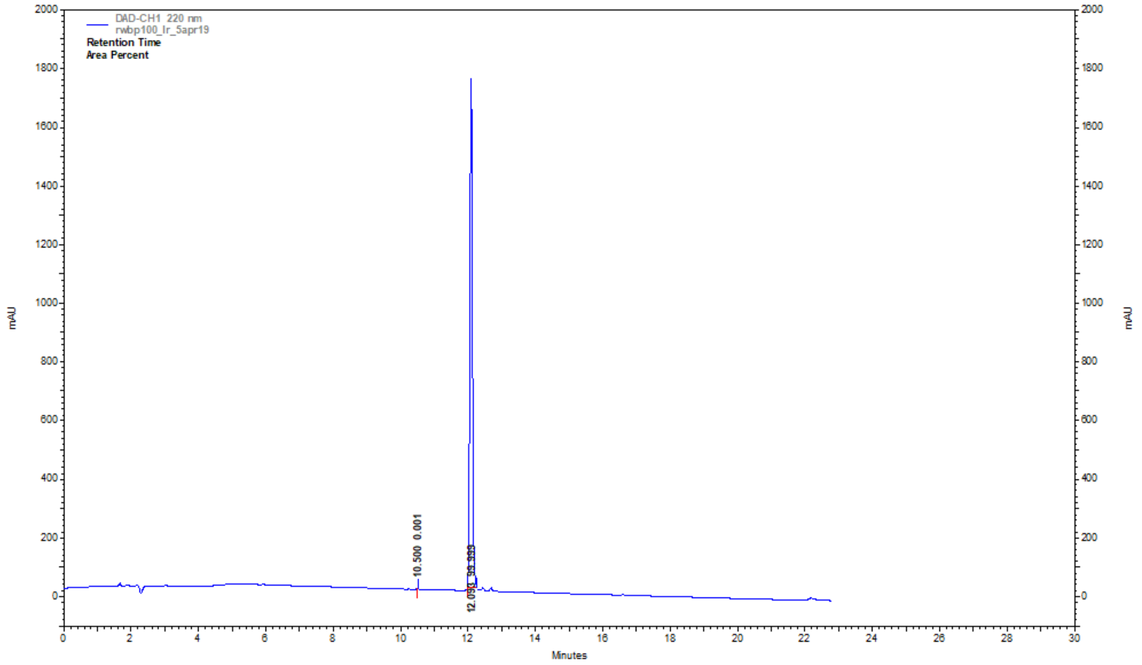

Figure S4. RP-HPLC chromatogram for peptide RW-BP100, after purification; gradient elution from 1 to 100% ACN in 0.05% aqueous TFA at 1 mL/min flow rate, for 30 min, on a C-18 column (150 × 4.6 mm ID and 5 µm pore size); detection at 220 nm.

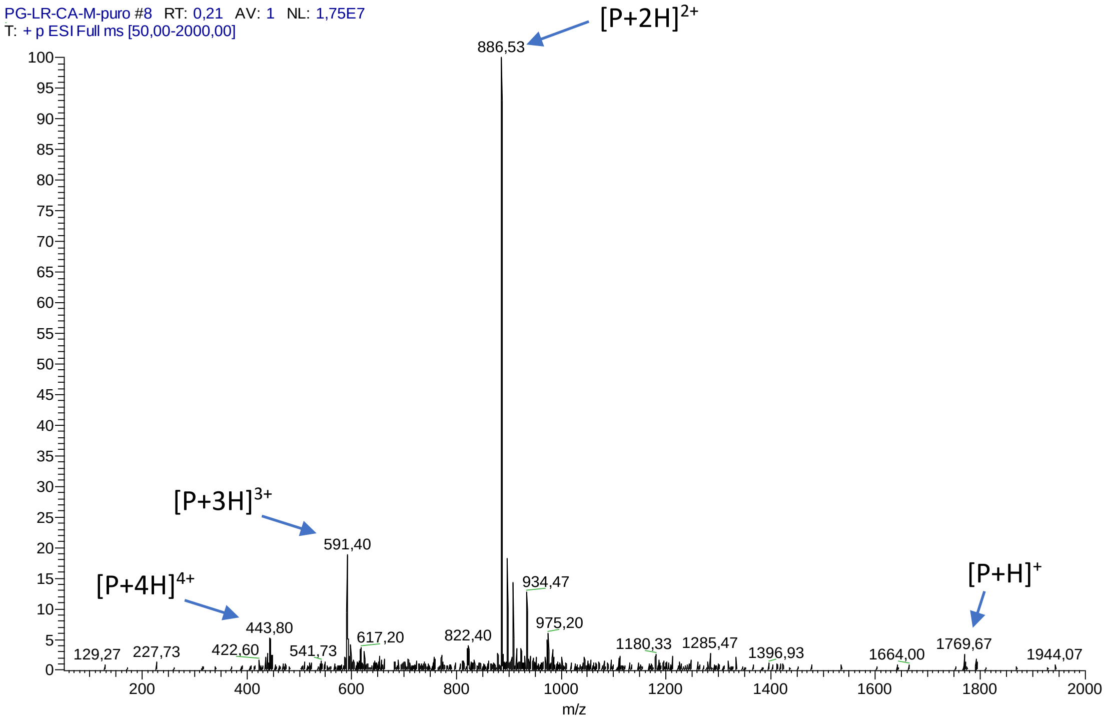

Figure S5. Mass spectrum (ESI-IT, positive mode) of peptide CA-M (MW=1769.2 Da), showing the quasi-molecular ion ([P+H]$^{+}$), the di-protonated ([P+2H]$^{2+}$, base peak), the tri-protonated ([P+3H]$^{3+}$), and the tetra-protonated ([P+4H]$^{4+}$) ions of the target peptide (P).

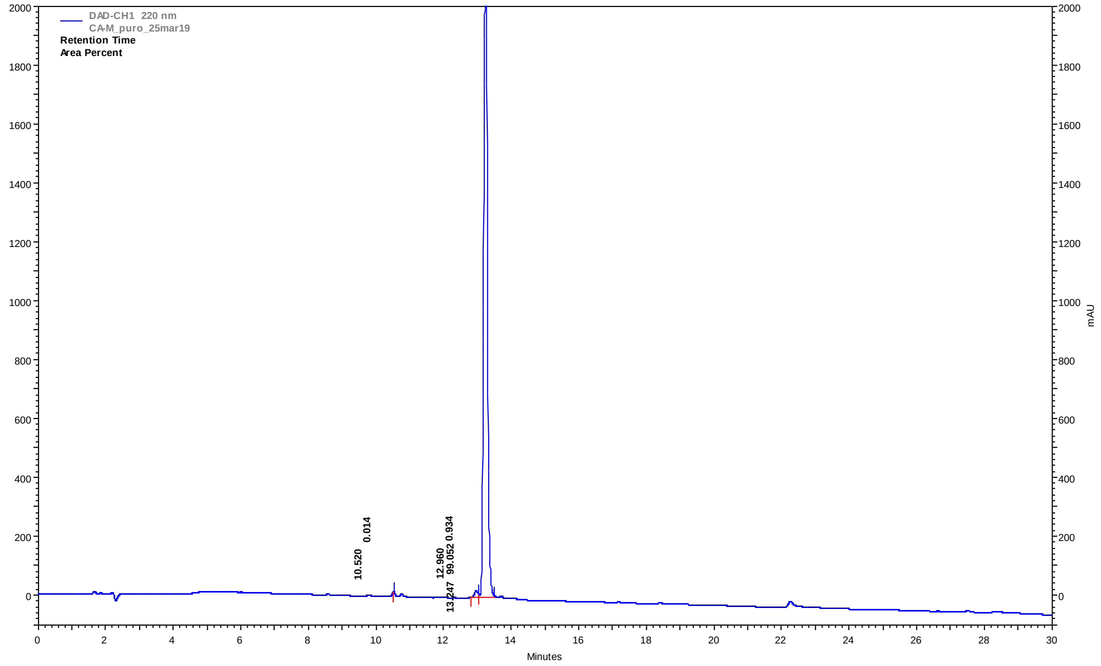

Figure S6. RP-HPLC chromatogram for peptide CA-M after purification; gradient elution from 1 to 100% ACN in 0.05% aqueous TFA at 1 mL/min flow rate, for 30 min, on a C-18 column (150 × 4.6 mm ID and 5 μm pore size); detection at 220 nm.

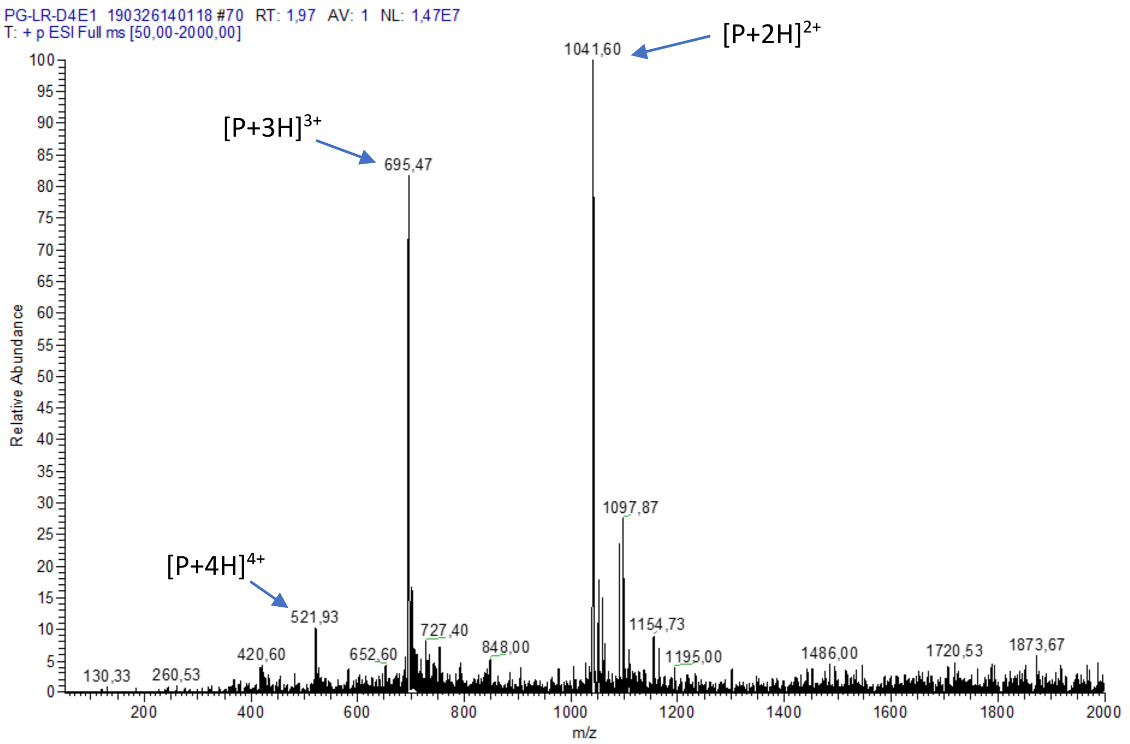

Figure S7. Mass spectrum (ESI-IT, positive mode) of peptide D4E1 (MW=2079.4 Da), highlighting the di-protonated ($[P+2H]^{2+}$, base peak), tri-protonated ($[P+3H]^{3+}$) and tetra-protonated ($[P+4H]^{4+}$) ions of the target peptide (P).

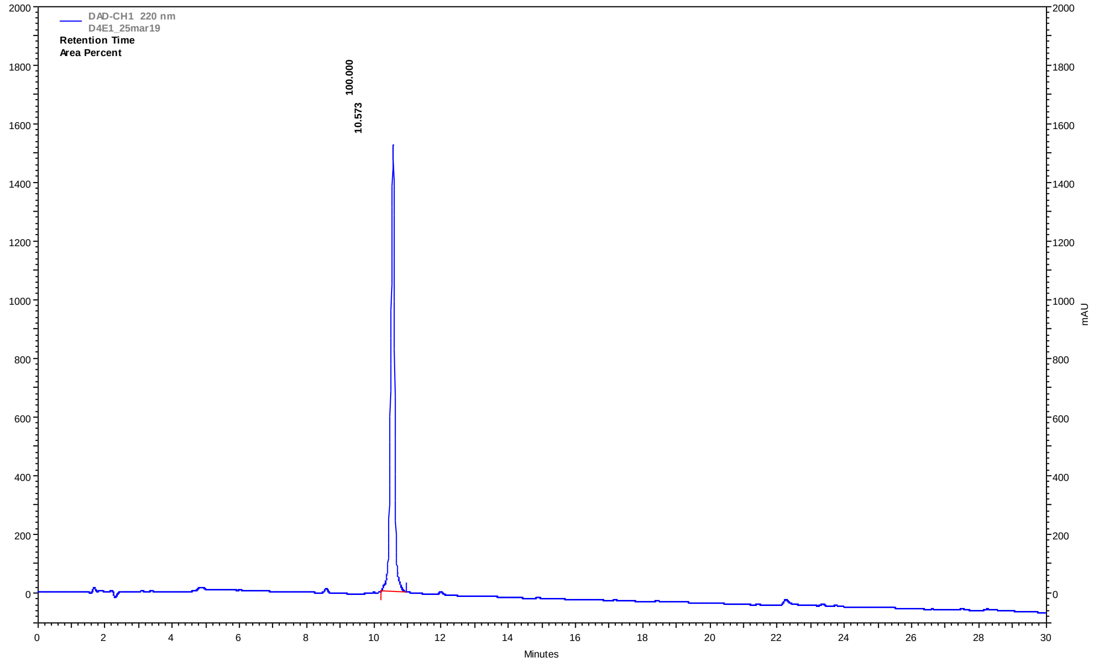

Figure S8. RP-HPLC chromatogram for peptide D4E1, after purification; gradient elution from 1 to 100% ACN in 0.05% aqueous TFA at 1 mL/min flow rate, for 30 min, on a C-18 column (150 × 4.6 mm ID and 5 µm pore size); detection at 220 nm.

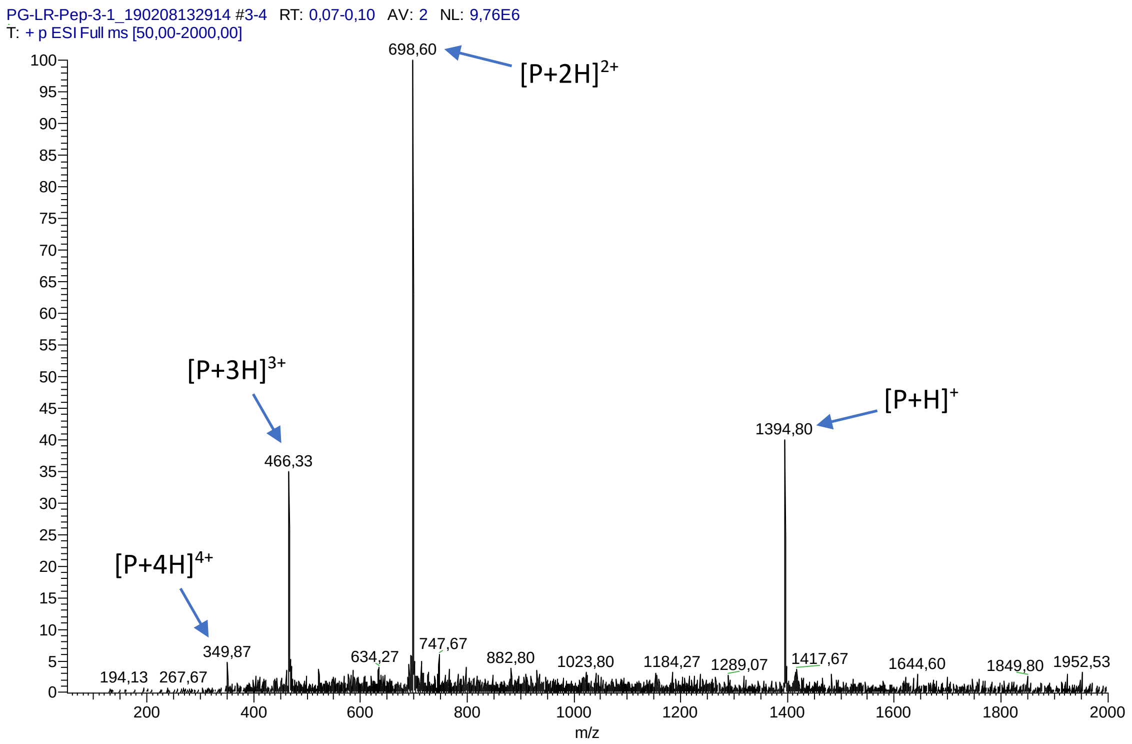

Figure S9. Mass spectrum (ESI-IT, positive mode) of peptide 3.1 (MW=1393.9 Da), showing the quasi-molecular ($[P+H]^+$, base peak), di-protonated ($[P+2H]^{2+}$), tri-protonated ($[P+3H]^{3+}$) and tetra-protonated ($[P+4H]^{4+}$) ions of the target peptide (P).

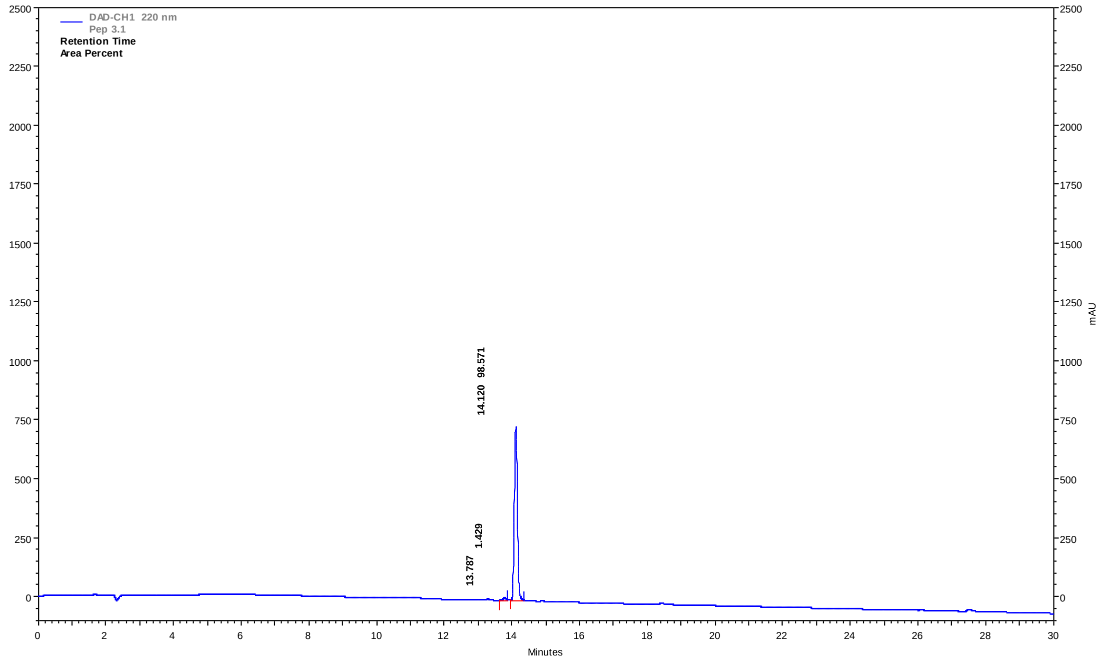

Figure S10. RP-HPLC chromatogram for peptide 3.1, after purification; gradient elution from 1 to 100% ACN in 0.05% aqueous TFA at 1 mL/min flow rate, for 30 min, on a C-18 column (150 × 4.6 mm ID and 5 μm pore size); detection at 220 nm.

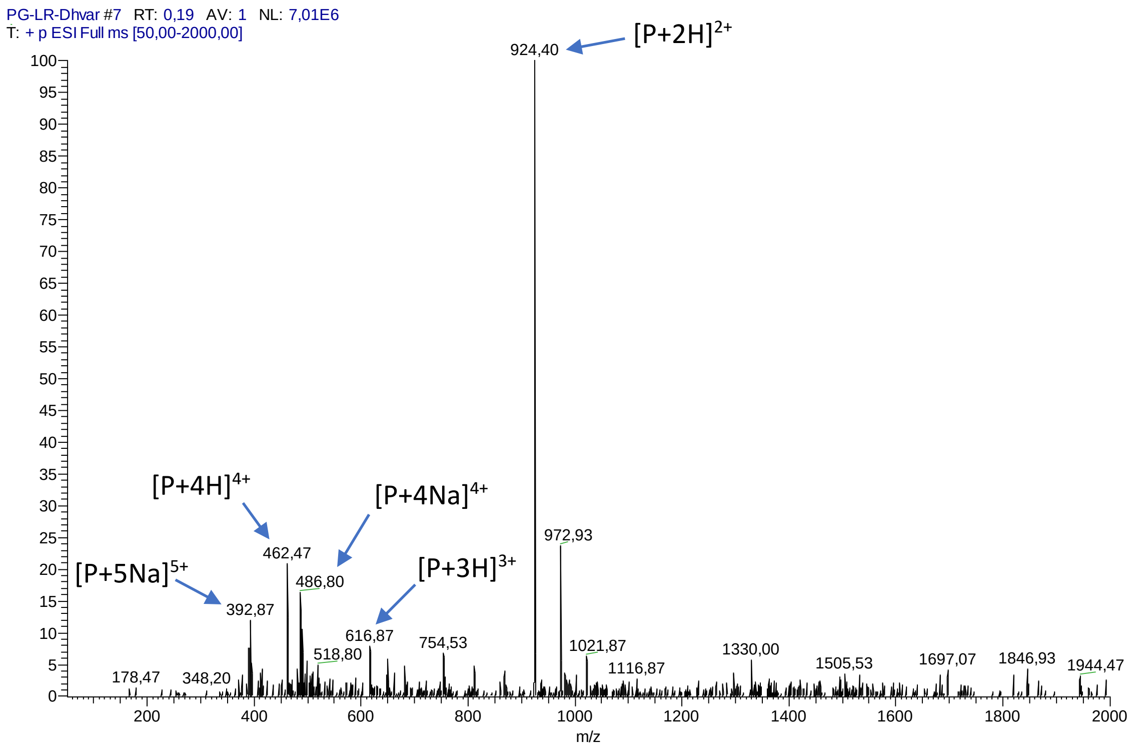

Figure S11. Mass spectrum (ESI-IT, positive mode) of peptide Dhvar-5 (MW=1845.3 Da), highlighting the di-protonated ion ($[P+2H]^{2+}$, base peak), the tri-protonated ion ($[P+3H]^{3+}$), the tetra-protonated ion ($[P+4H]^{4+}$) and its sodium adduct ($[P+4Na]^{4+}$), and the penta-sodium adduct ($[P+5Na]^{5+}$) of the target peptide (P).

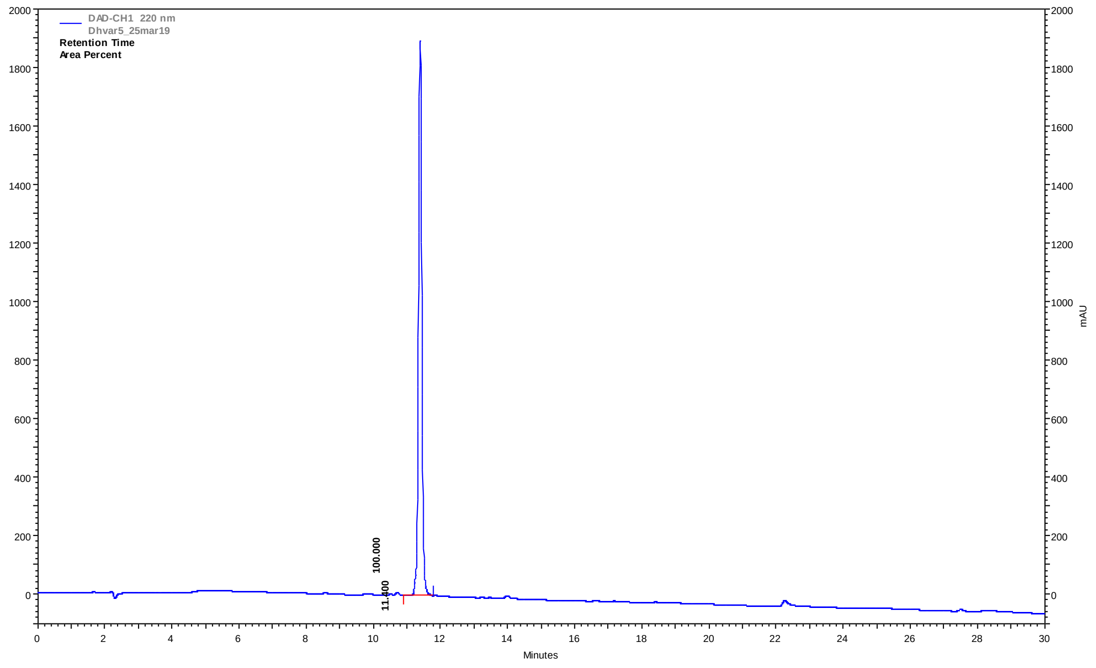

Figure S12. RP-HPLC chromatogram for peptide Dhvar-5, after purification; gradient elution from 1 to 100% ACN in 0.05% aqueous TFA at 1 mL/min flow rate, for 30 min, on a C-18 column (150 × 4.6 mm ID and 5 µm pore size); detection at 220 nm.

Table S1. RP-HPLC data on test peptides.

Peptide	Purity (%)	Retention time (min)
BP100	100	11.31
RW-BP100	100	12.09
CA-M	99.0	13.25
D4E1	100	10.57
3.1	98.6	14.12
Dhvar-5	100	11.40

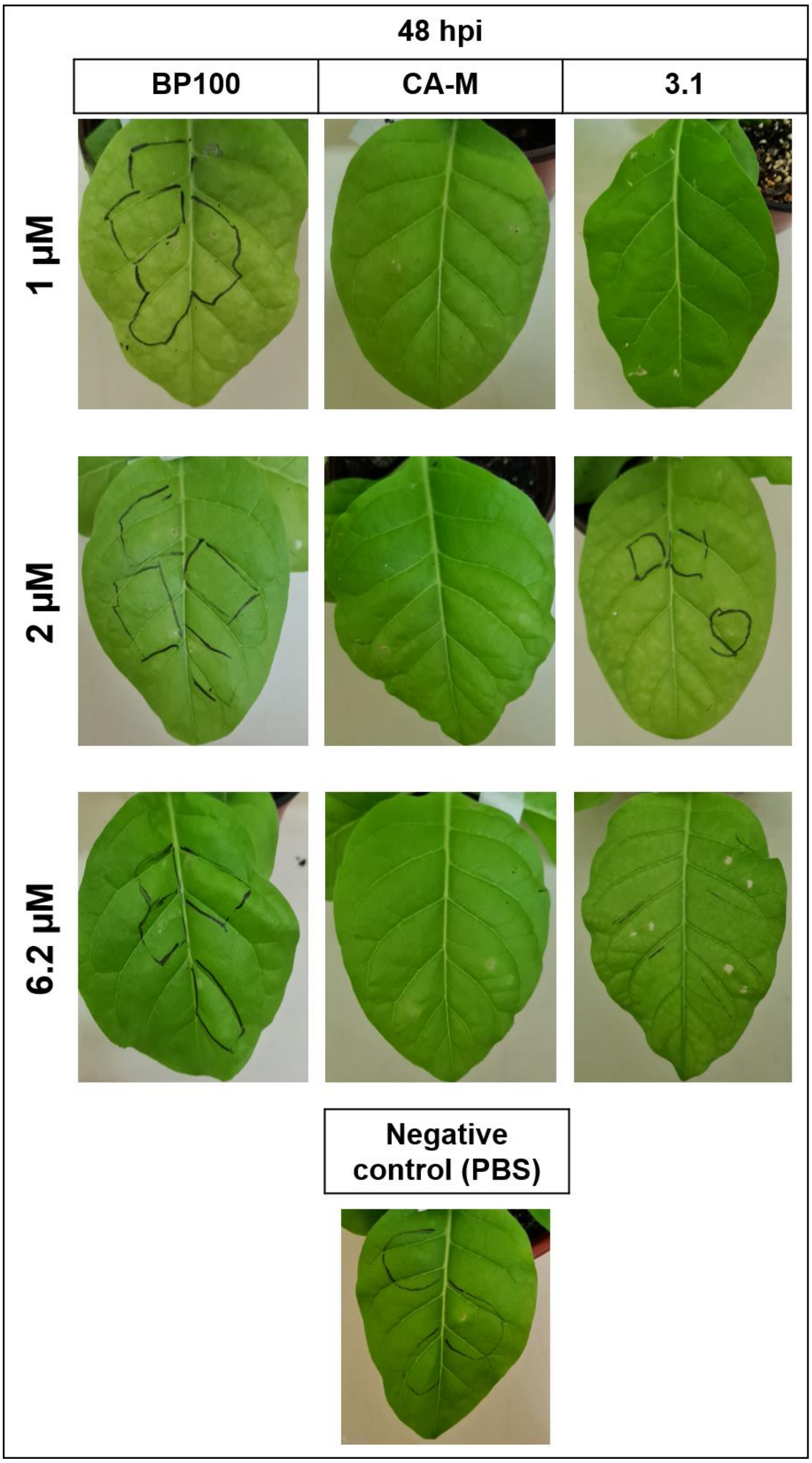

Figure S13. Negative controls of the HR test in tobacco leaves. Only a solution of PBS and AMPs (BP100, CA-M and 3.1) at final concentration were used to infiltrate tobaccos (1 mL). Negative control with PBS was also used. Images were recorded after 48 hours post infiltration (hpi).

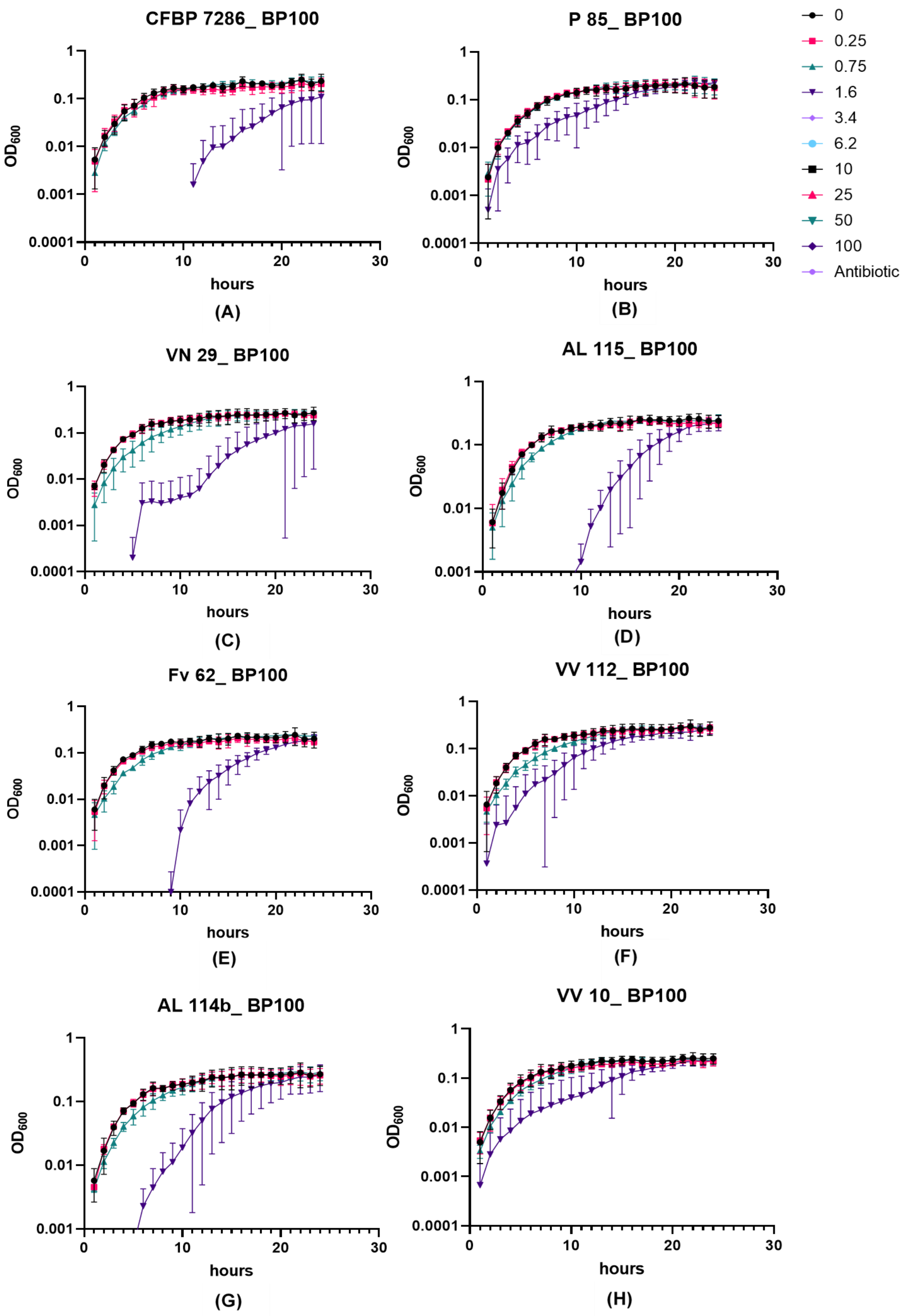

Figure S14. Growth curves of Psa strains (A) CFBP 7286, (B) P85, (C) VN29, (D) AL115, (E) Fv62, (F) VV112, (G) AL114b and (H) VV10 treated with BP100 AMP at 0, 0.25, 0.75, 1.6, 3.4, 6.2, 10, 25, 50 and 100 µM, monitored by absorbance measurement at 600 nm, during 24 hours. The antibiotic chlortetracycline at 25 µM was used as a positive control.

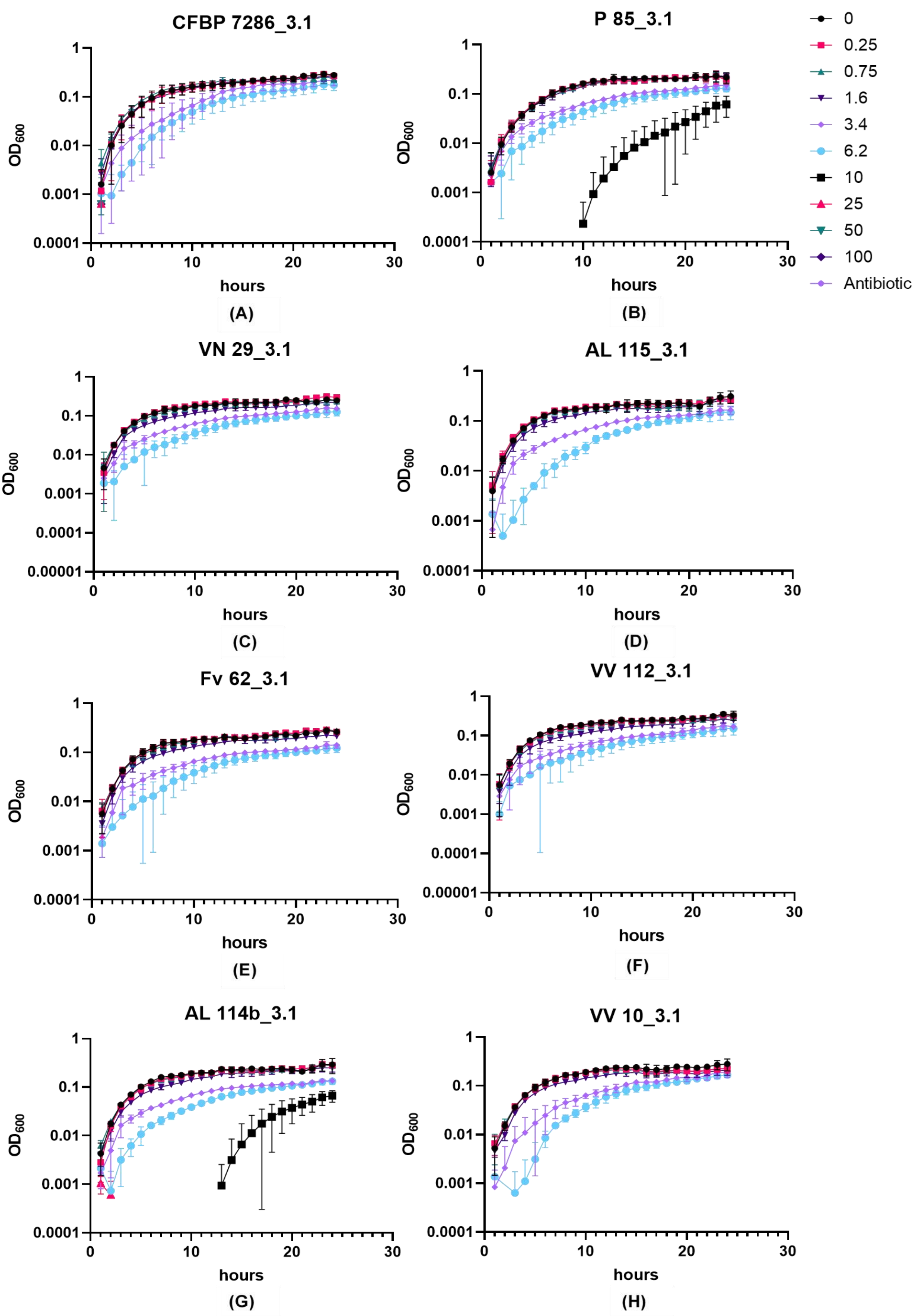

Figure S15. Growth curves of Psa strains (A) CFBP 7286, (B) P85, (C) VN29, (D) AL115, (E) Fv62, (F) VV112, (G) AL114b and (H) VV10 treated with 3.1 AMP at 0, 0.25, 0.75, 1.6, 3.4, 6.2, 10, 25, 50 and 100 µM, monitored by absorbance measurement at 600 nm, during 24 hours. The antibiotic chlortetracycline at 25 µM was used as a positive control.

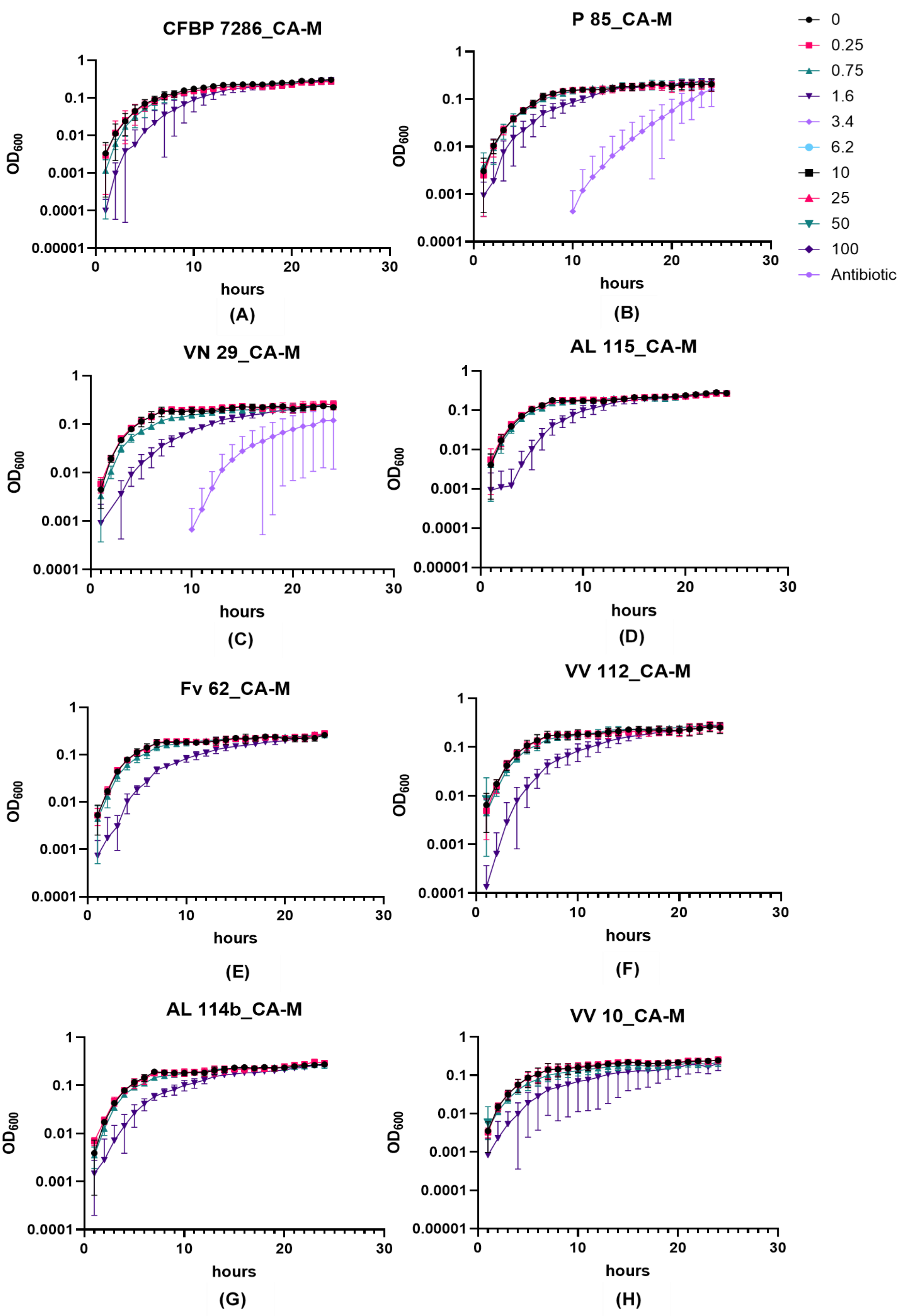

Figure S16. Growth curves of Psa strains (A) CFBP 7286, (B) P85, (C) VN29, (D) AL115, (E) Fv62, (F) VV112, (G) AL114b and (H) VV10 treated with CA-M AMP at 0, 0.25, 0.75, 1.6, 3.4, 6.2, 10, 25, 50 and 100 μM, monitored by absorbance measurement at 600 nm, during 24 hours. The antibiotic chlortetracycline at 25 μM was used as a positive control.

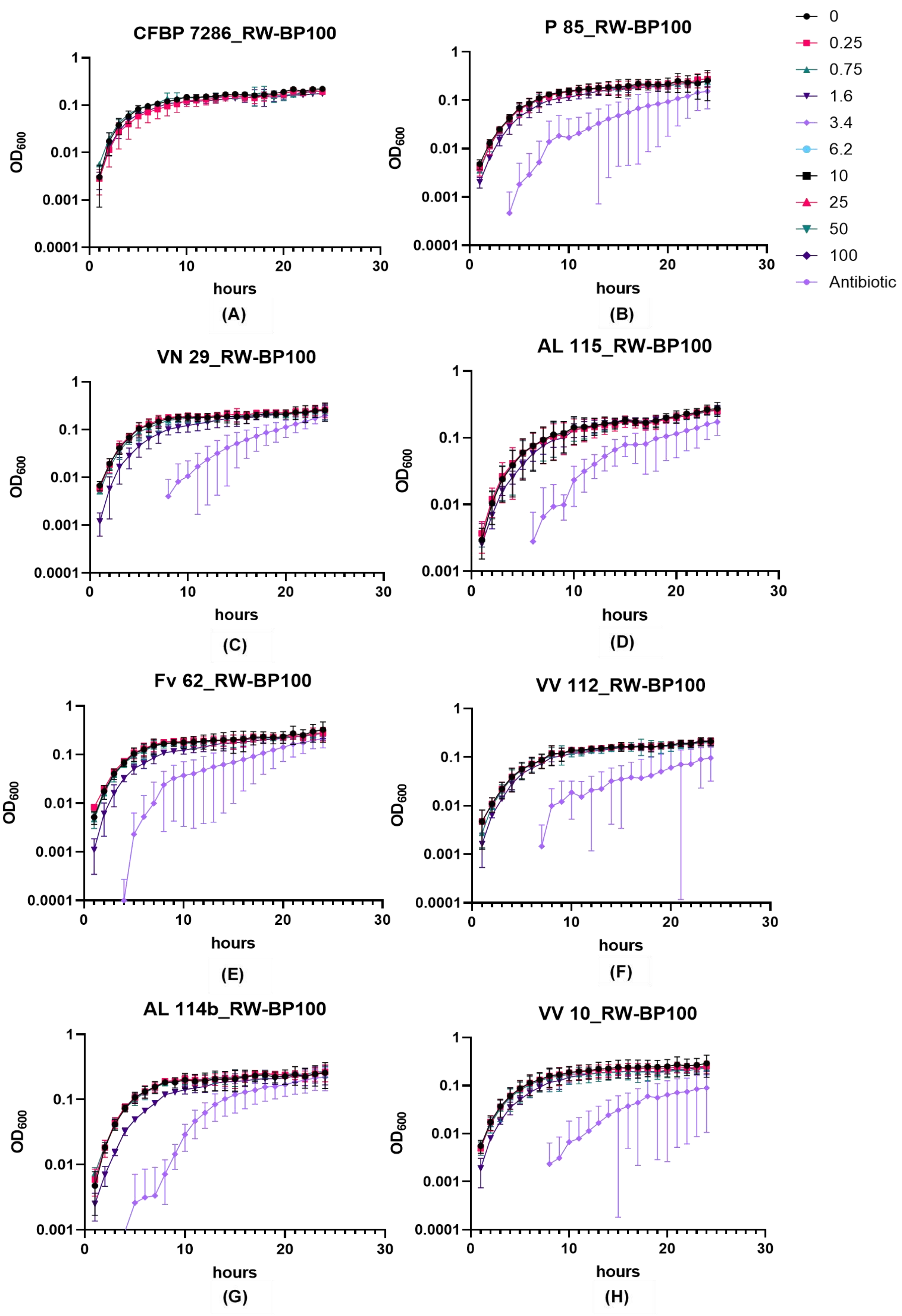

Figure S17. Growth curves of Psa (A) CFBP 7286, (B) P85, (C) VN29, (D) AL115, (E) Fv62, (F) VV112, (G) AL114b and (H) VV10 treated with RW-BP100 AMP at 0, 0.25, 0.75, 1.6, 3.4, 6.2, 10, 25, 50 and 100 µM, monitored by absorbance measurement at 600 nm, during 24 hours. The antibiotic chlortetracycline at 25 µM was used as a positive control.
